# Microarray-Based Identification of Differentially Expressed Genes in Intracellular *Brucella abortus* within RAW264.7 Cells

**DOI:** 10.1371/journal.pone.0067014

**Published:** 2013-08-07

**Authors:** Mingxing Tian, Jing Qu, Xiangan Han, Min Zhang, Chan Ding, Jiabo Ding, Guanghua Chen, Shengqing Yu

**Affiliations:** 1 Shanghai Veterinary Research Institute, Chinese Academy of Agricultural Science, Shanghai, P. R. China; 2 College of Veterinary Medicine, Yangzhou University, Yangzhou, P. R. China; 3 China Institute of Veterinary Drug Control, Beijing, P. R. China; Universite de la Mediterranee, France

## Abstract

*Brucella spp*. is a species of facultative intracellular Gram-negative bacteria that induces abortion and causes sterility in domesticated mammals and chronic undulant fever in humans. Important determinants of *Brucella*’s virulence and potential for chronic infection include the ability to circumvent the host cell’s internal surveillance system and the capability to proliferate within dedicated and non-dedicated phagocytes. Hence, identifying genes necessary for intracellular survival may hold the key to understanding *Brucella* infection. In the present study, microarray analysis reveals that 7.82% (244/3334) of all *Brucella abortus* genes were up-regulated and 5.4% (180/3334) were down-regulated in RAW264.7 cells, compared to free-living cells in TSB. qRT-PCR verification further confirmed a >5-fold up-regulation for fourteen genes. Functional analysis classified *araC*, *ddp*, and *eryD* as to partake in information storage and processing, *alp*, *flgF* and *virB9* to be involved in cellular processes, *hpcd* and *aldh* to play a role in metabolism, *mfs* and *nikC* to be involved in both cellular processes and metabolism, and four hypothetical genes (*bruAb1_1814*, *bruAb1_0475*, *bruAb1_1926*, and *bruAb1_0292*) had unknown functions. Furthermore, we constructed a *B. abortus* 2308 mutant Δ*ddp* where the *ddp* gene is deleted in order to evaluate the role of *ddp* in intracellular survival. Infection assay indicated significantly higher adherence and invasion abilities of the Δ*ddp* mutant, however it does not survive well in RAW264.7 cells. *Brucella* may survive in hostile intracellular environment by modulating gene expression.

## Introduction

*Brucella spp*. is a species of facultative, intracellular, Gram-negative bacteria that induces abortion and causes sterility in domesticated mammals and chronic undulant fever in humans [1]–[2]. *Brucella* has no classical virulence factors including exotoxins, cytolysins, capsules, fimbria, plasmids, lysogenic phages, drug resistant forms, antigenic variations, or endotoxic lipopolysaccharide (LPS) molecules [3]. Rather, *Brucella* pathogenicity owes to its capability to survive and proliferate within dedicated and non-dedicated phagocytes [4]–[5]. Although the pathogenic mechanisms of *Brucella* are not well understood, these bacteria often alter normal host functions to escape immune surveillance. Successful strategies for intracellular survival include inhibition of host cell apoptosis [6], survival in acidified membrane-bound vesicles [7], and prevention of phagosome-lysosome fusion [8]–[9]. Thus, the identification of *Brucella* genes necessary for intracellular survival is crucial to elucidate the infectious process in order to control of brucellosis.

To date, four major *Brucella* virulence factors that aid invasion and survival in host cells, namely LPS, the type-IV secretion system (T4SS), the BvrR/BvrS two-component regulatory system, and cyclic β-1,2-glucan, have been studied to a great extent [10]. Additional factors, such as the helix-turn-helix-type quorum sensing-dependent transcriptional regulator (*vjbR*), superoxide dismutase [Cu-Zn] (*sodC*), RNA binding protein host factor (*hfq*), 41 kDa surface protein (*ugpB*), and heat shock protein DnaK (*dnaK*), also reportedly affect *Brucella* invasion and replication capacity in host cells. However, the exact mechanisms by which *Brucella* forms replication niches in the endoplasmic reticulum remain unclear. Therefore, analyzing the changes in *Brucella* gene expression in intracellular environments will render better understanding of its pathogenesis.

Microarray analysis is a high-throughput screening method to simultaneously measure the expression levels of a large number of genes or to genotype multiple genomic regions. Eskra et al. [11] utilized microarray technology to identify over 140 differentially expressed genes in RAW264.7 cells (a murine macrophage cell line) when they were infected by *B. abortus*. Viadas et al. [12] performed whole-genome microarray analysis using *B. abortus* RNA obtained from wild-type and *Brucella* virulence related protein R (*bvrR*) mutant cells and identified a total of 127 differentially expressed genes in the *bvrR* mutants. However, microarray analysis of the gene expression profile between intracellular versus free-living *Brucella* has not yet been reported.

In the present study, a microarray assay was used to identify genes differentially expressed in *Brucella* within RAW264.7 cells and free-living bacteria in tryptic soy broth (TSB) medium. The results show that 7.82% of *Brucella* genes were up-regulated and 5.40% were down-regulated. Real-time quantitative reverse transcription-PCR (qRT-PCR) analysis further verified that the levels of 14 *Brucella* genes were up-regulated more than 5-fold. The microarray data suggest the possibility that *Brucella* survives within macrophages by modulating the expression of a series of genes in order to adapt to an intracellular environment.

## Results

### RNA Quality Analysis

RNA integrity was assessed by electrophoresis on a denaturing agarose gel and purity and concentration were measured using the NanoDrop ND-1000 spectrophotometer. Electrophoresis showed three distinct bands of 23S, 16S and 5S rRNA, indicating that the RNA was intact. Spectrophotometric RNA analysis revealed an OD_260_/OD_280_ ratio of >1.8, indicating superior quality of the RNA samples suitable for the microarray analysis.

### Determination of Differentially Expressed Genes

**Figure 1:**
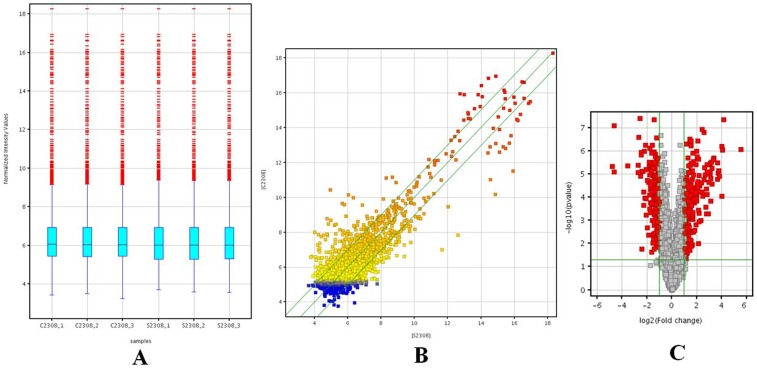
Quality assessment of gene data and differential gene expression screening. A: A box plot was used to compare the distributions of the intensities from all samples. The distributions of log_2_ ratios among samples are nearly the same; B: A scatter plot was used to assess gene expression variation (or reproducibility) between the two groups. The genes above the top green line and below the bottom green line indicate a >2.0-fold change in expression between the two groups; C: Volcano Plots were used to visualize differential expression between the two groups. The vertical lines correspond to 2.0-fold increase and decrease, whereas the horizontal line represents a p-value of 0.05. The red dot in the plot represents differentially expressed genes with statistical significance. “S2308” refers to the groups of free-living bacteria in TSB, “C2308” refers to the groups of intracellular bacteria.

Raw gene data were obtained by scanning the gene chips. Normalization and processing of the raw gene data were performed using the GeneSpring GX v11.5.1 software package (Agilent Technologies, Inc.). Afterward, genes with at least four of six samples flagged as “present” or “marginal” were chosen for screening of differentially expressed genes. The quality of gene data was assessed using box and scatter plots. The box plot was used to compare the intensity distributions of all samples. The distributions of log_2_ ratios among the samples were similar (Fig. 1A). The scatter plot was used to assess gene expression variation (or reproducibility) between the two groups. In Fig. 1B, the genes above the top green line and below the bottom green line indicated a >2.0-fold change between the two compared groups. Volcano Plot filtering was performed to identify statistically significant differentially expressed genes between the two groups. A change in gene expression was considered statistically significant if the fold change was >2.0 and the p-value was <0.05 (Fig. 1C). The microarray analysis show that 244 genes (7.82%, 244/3334) are up-regulated and 180 (5.40%, 180/3334) are down-regulated (a complete list of the differentially expressed genes between intra-RAW264.7 versus free-living *B. abortus* in TSB is shown in Table S1).

### Heat Map and Hierarchical Clustering for Differentially Expressed Genes

**Figure 2:**
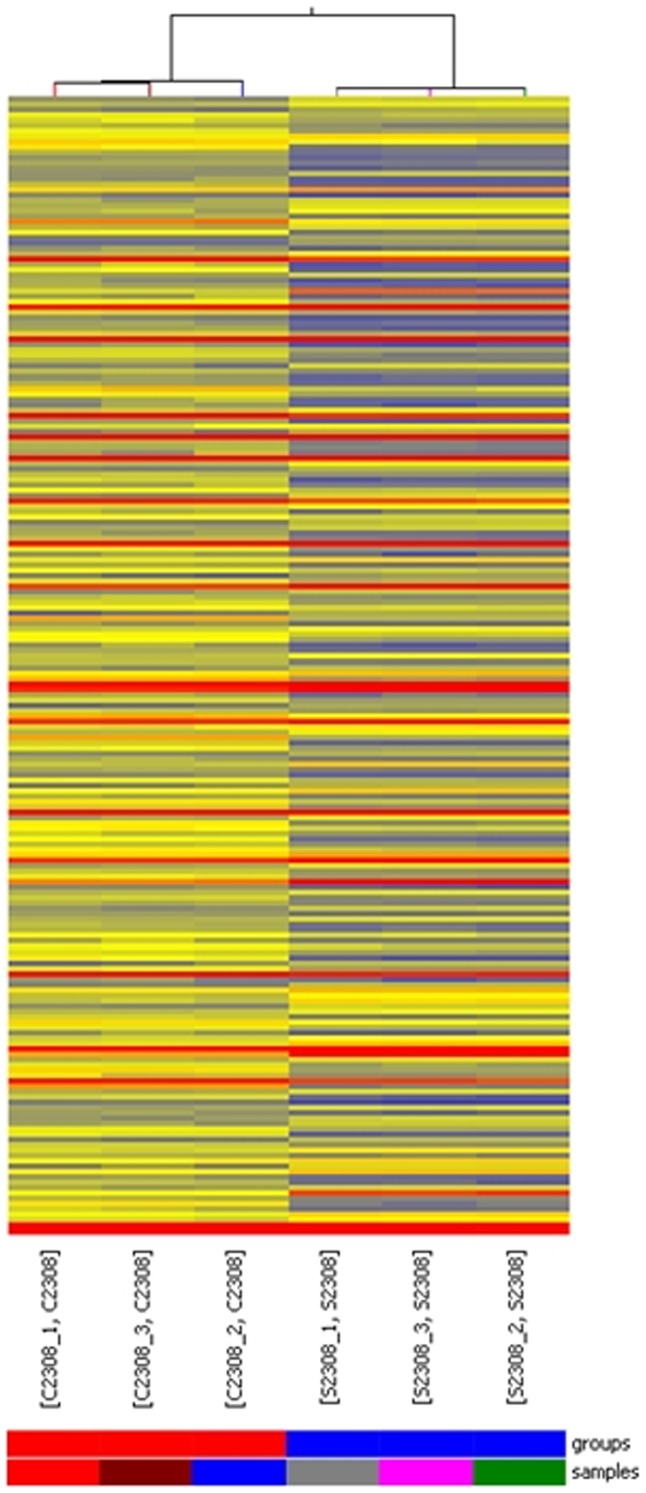
Heat map and hierarchical clustering. Hierarchical clustering was performed based on all differentially expressed gene data. The results of hierarchical clustering on conditions show distinguishable gene expression profiling between samples. “Red” indicates high relative expression, and “blue” indicates low relative expression. “S2308” refers to the groups of free-living bacteria in TSB, “C2308” refers to the groups of intracellular bacteria.

Hierarchical clustering is one of the simplest and most widely used clustering techniques for analyzing gene expression data. Cluster analysis arranges samples into groups based on their expression levels to elucidate possible relationships among samples. In the study, hierarchical clustering was performed based on all target genes values. Our experiment consisted of six different samples and the results of hierarchical clustering regarding various conditions show distinguishable gene expression profiling between samples (Fig. 2).

### Pathway Analysis for Differentially Expressed Genes

**Figure 3:**
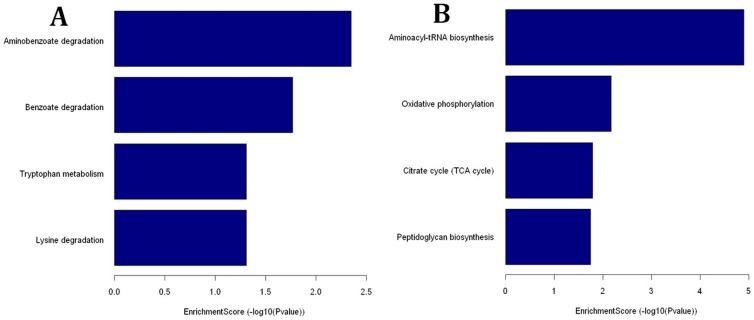
Significant pathway of differentially expressed genes. “P value” indicates the enrichment p-value of the Pathway ID determined by the Fisher's exact test. “Enrichment_Score” indicates the enrichment score value of the Pathway ID, which equals -log_10_ (p-value). A: Significant pathways of up-regulated genes; B: Significant pathway of down-regulated genes.

Pathway analysis for the differentially expressed genes was performed according to the latest KEGG database, which showed a significant enrichment of differentially expressed genes as determined by Fisher’s exact test p-values (cut-off limit, p-value ≤0.05). The up-regulated genes mostly occurred within four biological pathways: aminobenzoate degradation, benzoate degradation, lysine degradation, and tryptophan metabolism (Fig. S1-A), and the down-regulated genes were enriched in four others: aminoacyl-tRNA biosynthesis, oxidative phosphorylation, peptidoglycan biosynthesis, and citrate cycle (tricarboxylic acid (TCA) cycle) (Fig. S1-B). P-value-based enrichment scores reveal aminobenzoate degradation and aminoacyl-tRNA biosynthesis as the most significantly enriched pathways in the up-regulated and down-regulated genes, respectively (Fig. 3).

### qRT-PCR Verification and Functional Categories of Differentially Expressed Genes

Among the 244 up-regulated and 180 down-regulated genes identified by the microarray analysis, 33 up-regulated and 7 down-regulated genes, which were differentially expressed by >5-fold based on the microarray, were subjected to further validation. qRT-PCR confirmed 32 up-regulated and 3 down-regulated genes, of which 14 genes were up-regulated by >5-fold (Table 1). We presume that these genes would be important to *Brucella* survival within RAW264.7 cells. Of the 14 up-regulated gene products, AraC transcriptional regulator (AraC, BruAb2_1128), DnaA domain protein (Ddp, BruAb1_0593), and erythritol transcriptional regulator (EryD, BruAb2_0365) are involved in cell information storage and processing pathways; alkaline phosphatase (Alp, BruAb1_1205), flagellar basal body rod protein (FlgF, BruAb2_0126), and type IV secretion system protein VirB9 (VirB9, BruAb2_0061) are involved in cellular processes; homoprotocatechuate 2,3-dioxygenase (Hpcd, BruAb2_1096) and the aldehyde dehydrogenase family of proteins (ALDH, BruAb1_0205) are involved in metabolic pathways; major facilitator family transporters (MFS, BruAb2_0692) and nickel transporter permease (NikC, BruAb2_0430) are involved in both cellular processes and metabolic pathways; and four hypothetical proteins, encoded by BruAb1_1814, BruAb1_0475, BruAb1_1926, and BruAb1_0292, have unknown functions.

**Table 1:** Intracellular transcriptional level of *Brucella* genes obtained by qRT-PCR and analysis of gene functional characteristics.

B. abortus ORF^a^	Products	Subcellular location^b^	Function group(COGs)^c^	2^−ΔΔCte^
BruAb2_1128	AraC family transcriptional regulator	unknown	COG2207/K	44.28
BruAb2_1096	homoprotocatechuate 2,3-dioxygenase	unknown	COG0346/E	28.63
BruAb1_1814	hypothetical protein	unknown	–d	26.86
BruAb1_0593	DnaA domain protein	unknown	COG0593/L	22.78
BruAb1_1205	phoA alkaline phosphatase	Outer Membrane	COG1785/P	18.24
BruAb2_0692	major facilitator family transporter	Outer Membrane	COG0477/GEPR	12.30
BruAb2_0126	flagellar basal body rod protein	Outer Membrane	COG1749/N	12.00
BruAb1_0205	aldehyde dehydrogenase family protein	Outer Membrane	COG1012/C	11.45
BruAb2_0430	nickel transporter permease	Outer Membrane; Extracellular	COG1173/EP	10.93
BruAb1_0475	hypothetical protein	Unknown	–	10.53
BruAb2_0061	type IV secretion system protein VirB9	Outer Membrane; Extracellular	COG3504/N	10.44
BruAb1_1926	hypothetical protein	Outer Membrane; Extracellular	–	8.30
BruAb1_0292	hypothetical protein	Unknown	–	8.20
BruAb2_0365	erythritol transcriptional regulator	Outer Membrane; Extracellular	COG2390/K	6.81
BruAb1_0291	hypothetical protein	Unknown	–	4.90
BruAb1_2124	protease	Outer Membrane; Extracellular	COG1214/O	4.86
BruAb1_1442	glycosy hydrolase family protein	Unknown	–	4.69
BruAb2_0772	pseudo	Outer Membrane	–	4.41
BruAb1_1305	hypothetical protein	Unknown	COG2510/S	4.25
BruAb2_0753	ABC transporter, periplasmic substrate-binding protein	Outer Membrane	COG0715/P	3.85
BruAb1_1068	hypothetical protein	Outer Membrane	COG0500/QR	3.56
BruAb1_0702	pdxA 4-hydroxythreonine-4-phosphate dehydrogenase	Unknown	COG1995/H	3.49
BruAb1_0052	hypothetical protein	Unknown	–	3.47
BruAb2_0572	renal dipeptidase family protein	Outer Membrane; Extracellular	COG2355/E	2.50
BruAb1_1551	hypothetical protein	outer membrane protein	COG3047/M	2.44
BruAb1_2148	YaeC family lipoprotein	Outer Membrane	COG1464/R	2.39
BruAb1_0016	enoyl-CoA hydratase	Unknown	COG1024/I	2.36
BruAb1_0603	hypothetical protein	Unknown	–	1.96
BruAb2_1009	methionine sulfoxide reductase A	Outer Membrane	COG0225/O	1.70
BruAb1_1381	pyridoxine 5′-phosphate synthase	Outer Membrane	COG0854/H	1.54
BruAb1_1330	sulfate ABC transporter sulfate-binding protein	Outer Membrane; Extracellular	COG1613/P	1.52
BruAb1_0427	glycyl-tRNA synthetase subunit beta	Outer Membrane; Extracellular	COG0751/J	1.51
BruAb2_0277	branched-chain amino acid ABC transporter, permease protein	Outer Membrane	COG0559/E	1.47
BruAb1_0505	processing protease	Outer Membrane	COG0612/R	1.38
BruAb1_1032	pseudo	Outer Membrane; Extracellular	–	1.36
BruAb1_0993	hypothetical protein	Unknown	–	1.30
BruAb1_0099	response regulator	Unknown	COG0784/T	0.66
BruAb1_1049	hypothetical protein	Unknown	–	0.59
BruAb2_0699	2-oxoisovalerate dehydrogenase E1 component, beta subunit	Outer Membrane	COG0022/C	0.33
BruAb2_0700	2-oxoisovalerate dehydrogenase E1 component, alpha subunit	Unknown	COG1071/C	0.29

a *B. abortus* ORFs listed are used locus tag of genes in *B. abortus* strain 9-941.

b Subcellular locations were predicted by the PSORTb v.3.0 server. Available: http://www.psort.org/psortb/index.html. Accessed 10 December 2012.

c Functional characterization of the proteins was predicted by the software COGnitor. Available: http://www.ncbi.nlm.nih.gov/COG/old/xognitor.html. Accessed 10 December 2012. Functional categories: (1) Information storage and processing: (J: Translation, ribosomal structure and biogenesis; K: Transcription; L: DNA replication, recombination and repair); (2) Cellular processes: (D: Cell division and chromosome partitioning; O: Posttranslational modification, protein turnover, chaperones; M: Cell envelope biogenesis, outer membrane; P: Inorganic ion transport and metabolism; T: Signal transduction mechanisms); (3) Metabolism: (C: Energy production and conversion; E: Amino acid transport and metabolism; F: Nucleotide transport and metabolism); (4) Poorly characterized: (R: General function prediction only; S: Function unknown).

d -: No related COG.

e Results are expressed as 2^−ΔΔCt^. Figures = 1 indicate that the gene is expressed similarly in both conditions, figures >1 indicate that the gene is over expressed in intracellular *Brucella*, and figures <1 indicate that the gene is expressed less in intracellular *Brucella*.

### The Δ*ddp* Mutant Efficiently Adheres and Invades RAW264.7 Cells

**Figure 4:**
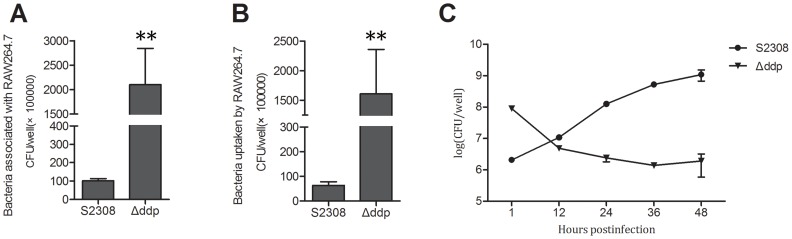
Infection assay. RAW264.7 cells were infected with Δ*ddp* mutant or wild type strain 2308 at a MOI 100∶1. A: The CFUs of total bacteria adherence to RAW264.7 cells. B: The CFUs of total bacteria invasion of RAW264.7 cells. C: Bacterial survival in RAW264.7 cells at 1 h, 12 h, 24 h, 36 h and 48 h p.i. All data are expressed as mean ± standard deviation and subjected to Student’s t-test, **, p≤0.01.

We assessed the ability of the Δ*ddp* mutant to adhere to and invade macrophages. RAW264.7 cells were infected with the Δ*ddp* mutant and its wild type counterpart S2308. Total bacteria associated with cells including adhesive and invasive bacteria were determined at 1 h post infection (p.i.). The results show that the Δ*ddp* mutant adheres and invades RAW264.7 cells more efficiently than S2308, shown by 20 times and 25 times higher colony forming units (CFUs) in the adherence and invasion assays, respectively (Fig. 4A–B).

### The Δ*ddp* Mutant Fails to Survive in RAW264.7 Cells

We assessed whether the Δ*ddp* gene is important in *Brucella*’s intracellular survival, RAW264.7 cells were infected with Δ*ddp* mutant or its wild type counterpart S2308 strain at a multiplicity of infection (MOI) 100∶1. Recovered bacteria were determined at 1 h, 12 h, 24 h, 36 h, and 48 h p.i. CFU of recovered Δ*ddp* mutant was significantly higher than that of the wild type strain at 1 h p.i., but drops sharply at 12 h p.i., and remains low until 48 h p.i. (Fig. 4C). The results reveal that the *ddp* gene plays an important role in *B. abortus* survival in murine macrophages.

## Discussion

The Agilent *B. abortus* bv. 1 str. 9-941 microarray is a broad-view assay that includes 3,334 established and predicted genes and transcripts. Here, our microarray analysis shows that 7.82% of the genomic *B. abortus* genes are up-regulated and 5.40% are down-regulated in intracellular *Brucella* in RAW264.7 cells compared to free-living *Brucella* in TSB.

The biological pathway analysis of differentially expressed genes reveals that the up-regulated genes mainly belong to aminobenzoate degradation, benzoate degradation, lysine degradation, and tryptophan metabolism pathways, indicating that *Brucella* may utilize several metabolic substances to provide necessary nutrients or energy for survival in hostile intracellular environments. Aminobenzoate is an intermediate derived from tryptophan and other indole ring-containing compounds and its catabolism has been described in aerobic bacteria. Reportedly, *Pseudomonas sp.* can proliferate under anaerobic conditions with 2-aminobenzoate and nitrate as the sole carbon and energy sources, respectively [13]–[14]. Further, 2-aminobenzoate can be catalyzed by anthranilate-CoA ligase and 2-aminobenzoyl-CoA reductase to produce coenzyme A thioesters of benzoate (benzoyl-CoA), which then participates in the benzoate pathway [13]. Benzoate is the most common intermediate in anaerobic metabolism of aromatic compounds and it is activated to benzoyl-CoA. In the absence of molecular oxygen, the aromatic ring is first reduced prior to ring cleavage. Dearomatization of the benzene ring occurs upon a two-electron reduction of benzoyl-CoA to cyclohex-diene-1-carboxyl-CoA, which is eventually metabolized for acetyl-CoA via a complex catalytic process. Most studies of anaerobic benzoate degradation were carried out using the phototrophic bacterium *Rhodopseudomonas palustris* [15]–[16] and two denitrifying species, *Thauera aromatica* K172 and *Azoarcus evansii* [17]–[18]. In *Brucella spp*., it was hypothesized that intracellular *Brucella* may utilize metabolism intermediates of aromatic compounds, especially aminobenzoate and benzoate, as carbon and energy sources for survival in hostile intracellular environments. As for lysine degradation and tryptophan metabolism in mammals, lysine is metabolized to form acetyl-CoA, via an initial transamination with α-ketoglutarate. In bacteria, however, degradation of lysine yields cadaverine by decarboxylation [19]. In microorganisms, tryptophan is degraded by several different pathways, in which oxygenase-catalyzed reactions play an important role. In *Brucella*, the intermediates of lysine degradation and tryptophan metabolism may be utilized for intracellular survival to provide energy or nutrition.

Alternatively, in the present study, most of the down-regulated genes belong to oxidative phosphorylation, citrate cycle (TCA cycle), aminoacyl-tRNA biosynthesis and peptidoglycan biosynthesis pathways. The first two pathways suggest that *Brucella*’s ability to use energy is weakened by the oxidation of nutrients to produce adenosine triphosphate (ATP) in intracellular environment. In addition, down-regulation of genes in aminoacyl-tRNA biosynthesis and peptidoglycan biosynthesis suggests that *Brucella* may decrease amino acid utilization in hostile intracellular environments and transform the cell wall structure to adapt to the intracellular environment by reducing peptidoglycan biosynthesis.

Forty genes differentially expressed by >5-fold in the microarray analysis were further validated by qRT-PCR, which confirmed that 14 were indeed differentially expressed by >5-fold. Of these 14 genes, *araC*, *ddp*, and *eryD* are involved in information storage and processing pathways. AraC belongs to the AraC-like transcriptional regulator family, which is widespread among bacteria and regulate genes that have diverse functions, ranging from carbon metabolism to stress responses to virulence [20], [21]. In *Brucella spp*., more than 10 AraC-like transcriptional regulators have been identified by comparative genomic approaches, but their functions are still not well understood. In *B. abortus* S2308, the AraC-like transcriptional regulator DhbR is required for maximum expression of the 2,3-dihydroxybenzoic acid biosynthesis genes in response to iron deprivation [20]. A *Mycobacterium tuberculosis* strain with disrupted AraC homologue Rv1931c exhibits reduced survival both in macrophages and a murine infection model [21]. The *ddp* gene encodes the DnaA domain protein, which contains a helix-turn-helix motif that specifically interacts with the DNA region, but the exact function of the gene is unknown. The *eryD* gene is an erythritol transcriptional regulator located in the *eryABCD* locus and is necessary for erythritol catabolism [22]. The preferential growth of *B. abortus* in certain fetal material (placenta and chorion) appears to be due to the presence of erythritol in these tissues [23]. Anderson and Smith [23] revealed that erythritol is a main carbon and energy source for *B. abortus* in the presence of excess glucose and other potential metabolites. Interestingly, *B. abortus* vaccine strain S19 contains a 702-bp deletion in the *ery* locus, which affects two genes, *eryC* and *eryD* [24]–[25]; Nevertheless, Tn5 insertions and complementation analysis revealed that this deletion is not sufficient or required for virulence in a murine model [26].

Three of the up-regulated genes (*alp*, *flgF*, and *virB9*) are involved in cellular processes. In bacteria, Alp is located in the periplasmic space, outside the cell membrane. Although the purpose of this enzyme remains unclear, one hypothesis is that the Alp generates free phosphate groups, which is supported by the fact that Alp is usually produced by bacteria only during phosphate starvation [27]. Another hypothesis is that Alp dephosphorylates organic molecules, which may be important for bacterial uptake of organic compounds outside of cultures [28]. However, an *Escherichia coli* mutant lacking Alp survived well, because the mutant was unable to shut off Alp production [29]. Hence, we presumed that intracellular *Brucella* may generate additional free phosphate groups which are limited in intracellular environments. Conventionally, *Brucella* has been described as non-motile and nonflagellated; however, the presence of a sheathed flagellum was recently discovered in *Brucella melitensis* and it was involved in infection persistence in a murine model [30]–[31]. FlgF is an important component of flagella and was up-regulated in an intracellular environment, thus it might play an important role in chronic infection of animal models. Another gene, *virB9*, encodes a core structure of type IV secretion system (T4SS) in *Brucella* and is key to T4SS function. T4SS is an important *Brucella* virulence factors and is encoded by the *virB* operon, which consists of genes *virB1* to *virB12* [32]. The T4SS can transport effector molecules from *Brucella* into infected host cells, which is critical for *Brucella* survival and replication [32]. *Brucella virB9* over-expression in RAW264.7 cells confirms the function of T4SS.

Hpcd and ALDH are involved in metabolism. The enzyme 2,3-Hpcd is widely distributed in bacteria and represents a focal point in the degradation pathways of more complex aromatic compounds [33]–[34]. Hpcd catalyzes O_2_ cleavage and insertion of both oxygen atoms into the organic substrate to form α-hydroxy-δ-carboxymethyl cis-muconic semialdehyde [33], [35]. Following ring cleavage, the product is further metabolized to CO_2_ and the TCA intermediates, succinate and pyruvate [33]. ALDH is a family of polymorphic enzymes responsible for the oxidation of aldehydes to carboxylic acids [36]. We inferred that *Brucella* Hpcd and ALDH perform similar catalytic functions, as it may be important to *Brucella* intracellular survival to use intracellular organic compounds to synthesize essential energy or nutrition. Moreover, the *mfs* and *nikC* genes are involved in both cell processes and metabolism. MFS is a secondary carrier that transports small solutes in response to chemiosmotic ion gradients [37]–[38]. Through genome sequencing and biochemical and molecular analyses of dozens of families of transporters, MFS was found to occur ubiquitously in all classifications of living organisms [38], as well as the ATP-binding cassette (ABC) superfamily. Thus, *mfs* up-regulation may be important for *Brucella* survival in adapting to the ionic intracellular environment. *nikC* gene encodes a membrane protein analogous to the transport protein permease, In *E. coli*, uptake of nickel by the periplasmic binding protein-dependent transport system is required for the synthesis and activities of hydrogenase isoenzymes under anaerobic conditions [39]. *Brucella suis* possesses genes that encode counterparts that are highly similar to the five protein components of the *E. coli nikABCDE* operon [40]. In vitro expression of the *nikA* promoter-*gfp* fusion is activated by low oxygen or nickel levels and inactivation of *nikA* severely alters the activity of the nickel metalloenzyme urease in this bacterium [40]. However, *B. suis* and a *nikA* mutant displayed similar intracellular replication rates after infection of human monocytes [40].

In order to investigate whether the intracellularly induced genes function on the intracellular survival in RAW264.7 cells, we chose *ddp* from the list of identified genes to perform functional analysis. We constructed a mutant strain Δ*ddp* where the *ddp* gene is deleted. Δ*ddp* can adhere to and invade the RAW264.7 cells much more efficiently than its wild type counterpart S2308, but the CFU of recovered Δ*ddp* mutant rapidly decreases at 12 h p.i. and remains low. Morphologically cytopathic phenomena were observed in Δ*ddp* infected cells (data not shown), suggesting that the declined CFU of recovered Δ*ddp* may be due to the disruption of the cells. As reported previously, compared to the parental smooth strain, *B. abortus* rough mutant is uptaken by macrophages more efficiently and more cytopathic for macrophages, because the rough mutant enters macrophages through different portals than the smooth strains [41]. Cholesterol, ganglioside GM1, class A scavenger receptor, PI3-kanase and toll-like receptor 4 (TLR4) all contribute to smooth *Brucella* infection of murine macrophages [42]–[44]. Rough *Brucella* mutant invades macrophages through different portals and is destroyed and released prematurely as a result of macrophage death [42]. Whether the Δ*ddp* mutant enters and survives in RAW264.7 cells using similar mechanism as the rough *Brucella* mutant does need to be further investigated.

In conclusion, the present microarray analysis identified 14 genes that were up-regulated >5-fold in intracellular *Brucella* in RAW264.7 cells compared to free-living *Brucella* in TSB. The *ddp* gene of *Brucella* plays roles in the bacterial adherence to, invasion of, and survival in RAW264.7 cells. The present results present potentially important clues for elucidating the mechanisms behind *Brucella* intracellular survival. Further investigation is necessary to clarify the exact roles of the identified genes in *Brucella* intracellular survival.

## Methods

### Bacteria and Cell Line

*B. abortus* S2308 cells were obtained from the Chinese Veterinary Culture Collection Center (Beijing, China) and cultured in TSB (Difco, Franklin Lakes, NJ, USA) on a shaker platform (200 rpm) at 37°C for 24 h for sampling of the free-living bacteria or until mid log phase (0.6–0.8) for an infection assay. RAW264.7 cells were obtained from the American Type Culture Collection (Manassas, VA, USA) and maintained in Dulbecco’s modified Eagle’s medium (DMEM, Bio-West, Inc., Logan, UT, USA) supplemented with 10% fetal bovine serum (FBS, Bio-West, Inc), 100 IU/mL penicillin, and 100 µg/mL streptomycin (Sigma-Aldrich, Inc., St. Louis, MO, USA) at 37°C in an atmosphere of 5% CO_2_.

### Macrophage Infection

RAW264.7 cells were cultured to monolayer in six-well plates (Corning Inc., Corning, NY, USA) in DMEM complete medium. The cells were washed three times with antibiotic-free, FBS-free DMEM and then infected with *Brucella* at a MOI of 200∶1 as described previously [41]. After infection, the plates were centrifuged at 400×g for 5 min at 4°C and then incubated for 90 min at 37°C in 5% CO_2_. The cells were then washed three times with antibiotic- and FBS-free DMEM to remove extracellular bacteria and incubated in DMEM containing 100 µg/mL gentamicin for 1 h to kill the remaining extracellular bacteria. The medium was then replaced with DMEM supplemented with 3% FBS and 10 µg/mL gentamicin and the infected cells were incubated for 24 h at 37°C in 5% CO_2_ to sample the bacteria in RAW264.7 cells.

### Collection of Bacterial Samples for Transcriptional Analysis

Free-living *Brucella* cells were collected from a 24-h culture in TSB medium by centrifugation at 14000×g for 1 min. The collected bacteria were then suspended in 0.01 M phosphate-buffered saline (PBS, pH = 7.4) and centrifuged for RNA extraction. The intracellular *Brucella* cells were collected from the infected RAW264.7 cells. Briefly, the infected cells were washed three times with PBS, then immediately combined with 1 mL of RLT lysis buffer (Qiagen, Hilden, Germany), 1% β-mercaptoethanol, 1% (v/v) phenol, and 10% (v/v) ethanol per well [45]. Following incubation at 37°C for 10 min, the RAW264.7 cells were lysed and bacteria were collected by centrifugation at 14000×g for 10 min for RNA extraction.

### RNA Extraction and Purification

Total RNA was extracted from bacteria (1×10^8^ CFU) using TRIzol® RNA Isolation Reagent (Invitrogen, Carlsbad, CA, USA) according to the manufacturer’s protocol. Genomic DNA contamination was removed using the TURBO DNA-free kit (Ambion, Inc., Austin, TX, USA). RNA quantity and quality were evaluated using the NanoDrop ND-1000 spectrophotometer (NanoDrop Technologies, Inc., Wilmington, DE, USA). RNA integrity was assessed by standard denaturing agarose gel electrophoresis.

### RNA Labeling and Microarray Hybridization

Sample labeling and microarray hybridization were performed according to the Agilent One-Color Microarray-Based Gene Expression Analysis protocol (Agilent Technology, Inc., Santa Clara, CA, USA). The Agilent Quick Amp Labeling Kit (Agilent Technology, Inc.) was used for sample labeling. Briefly, 1 µg of total RNA from each sample was linearly amplified and fluorescently labeled with Cy3-deoxycytidine triphosphate. The labeled cRNAs were purified using the RNAeasy Mini Kit (Qiagen) and the concentrations and specific activities of the labeled cRNAs (pmol Cy3/µg cRNA) were measured using the NanoDrop ND-1000 spectrophotometer. The *B. abortus* bv. 1 str. 9-941 8×15 K Gene Expression Array (Agilent Technology, Inc.), which includes 3334 genes, was used for hybridization analysis. Each labeled cRNA sample (1 µg) was fragmented by adding 11 µL of 10× Agilent blocking agent and 2.2 µL of 25× fragmentation buffer, heated at 60°C for 30 min, and then combined with 55 µL of 2× gene expression hybridization buffer to dilute the labeled cRNA. Next, 100 µL of hybridization solution was dispensed into the gasket slide, which was then assembled to the gene expression microarray slide. The slides were incubated for 17 h at 65°C in a hybridization oven (Agilent Technologies, Inc.). To confirm the reproducibility of the gene expression data, six slides were used in the microarray analysis, three to measure gene transcription levels of free-living *Brucella* in TSB and three to determine genetic alterations of intracellular *Brucella* in RAW264.7 cells. The microarray analysis was performed using three biological replicates of each sample.

### Data Collection, Normalization, Analysis and Submission

The hybridized arrays were washed, fixed, and scanned using the Agilent DNA Microarray Scanner (catalog no.: G2505B, Agilent Technologies, Inc.). Agilent Feature Extraction software (version 10.7.3.1; Agilent Technologies, Inc.) was used to analyze the acquired array images. Raw signal intensities were normalized by the quantile method using the GeneSpring GX v11.5.1 software package (Agilent Technologies, Inc.), and low intensity genes were filtered. The gene data quality after filtering was assessed using box and scatter plots. Differentially expressed genes with statistical significance between the two groups were identified through Volcano Plot filtering (fold change ≥2.0, p-value ≤0.05). Hierarchical clustering was performed to distinguish gene expression patterns among the samples. Finally, based on the latest KEGG database (Kyoto Encyclopedia of Genes and Genomes, http://www.genome.jp/kegg), pathway analysis was employed to determine the roles of these differentially expressed genes in various biological pathways. All microarray data had been submitted to Gene Expression Ominibus Database, and the GEO accession number is GSE46459.

### qRT-PCR Analysis

Forty genes showed expression level changes of >5-fold in the microarray analysis and were further analyzed via qRT-PCR. The qRT-PCR primers were designed from *B. abortus* strains 2308 or 9-941 using Primer 3 software (http://frodo.wi.mit.edu/) (Table S2). *Brucella* and total RNA were prepared as described above. RNA (1 µg) was reverse transcribed into cDNA using random primers and Moloney murine leukemia virus reverse transcriptase (Promega Corp., Madison, WI, USA). The qRT-PCR reaction was performed using eight-strip tubes included with the Mastercycler® EP Realplex real-time PCR detection system (Eppendorf AG, Hamburg, Germany) in a total reaction volume of 20 µL containing 10 µL of Power SYBR Green PCR Master Mix (Applied Biosystems, Foster City, CA), 0.5 µL (10 µM) of each gene-specific sense and anti-sense primer pair, 1 µL of cDNA, and 8 µL of ddH_2_O. The cycling program included 2 min at 50°C, 10 min at 95°C, and 40 cycles of 95°C for 15 s and 60°C for 1 min. For each gene, qRT-PCR reactions were performed for three RNA samples isolated from three separate experiments and each reaction was performed in triplicate. Data were normalized using the the 2^−ΔΔCt^ method and the glyceraldehyde 3-phosphate dehydrogenase gene was used an as internal control. The final qRT-PCR data were presented as the means of three separate experiments.

### Functional Classification of Main Differentially Expressed Genes

To predict subcellular protein localization, the differentially expressed genes were analyzed using the online software PSORT Subcellular Localization Prediction Tool (version 3.0; http://www.psort.org/). Functional characterization of the proteins was predicted using COGnitor sequence comparison software (http://www.ncbi.nlm.nih.gov/COG/old/xognitor.html) by comparison of the sequence to the Clusters of Orthologous Group (COG) protein database (http://clovr.org/docs/clusters-of-orthologous-groups-cogs/), which is based on COG functional categories.

### Construction of *ddp* Gene Deleted Mutant and Infection Assay

A *ddp* gene deleted mutant strain Δ*ddp* was constructed as described [46]. Briefly, the upstream and downstream fragments of the *ddp* gene were amplified by PCR using two pairs of primers: Ddp-UF (gctctagagcgggtgggttgccattgtcag, *Xba*I site underlined)/Ddp-UR (tgcactgcagtgcaccaacagttccaaagatatc, *Pst*I site underlined) and Ddp-DF (tgcactgcagtgcaatggcagcatgacgaggcag, *Pst*I site underlined)/Ddp-DR (gctctagagcttgccgataataaccgctcc, *Xba*I site underlined). The fragments were then used as templates for a second round of PCR using primers Ddp-UF and Ddp-DR. The resulting fragment was inserted into pUC19-sacB plasmid at *Xba*I site to generate plasmid pUC19-SacB-Ddp, which was transformed electronically into. *B. abortus* S2308 competent cells.

Infection assay was performed on RAW 264.7 cells cultured in 24-well plate as described (46) to evaluate bacterial adherence, invasion, and survival in the cells. The cells were infected with Δ*ddp* mutant or wild-type S2308 at a MOI of 100∶1 for 1 h at 37°C. Intracellular survival of the mutant in the cells was monitored at 1 h, 12 h, 24 h, 36 h, and 48 h p.i. All assays were performed with triplicate wells and the results represent the average from three separated experiments.

### Statistical Analysis

All data were expressed as means ± standard deviations and subjected to Student’s t-test in the SPSS V17.0 software (SPSS Inc., Chicago, IL, USA) (*p*<0.05).

## Supporting Information

Figure S1 **Biological pathway of differentially expressed genes.** Yellow nodes are associated with down-regulated genes; orange nodes are associated with up-regulated or only whole dataset genes; green nodes have no significance in expression. The up-regulated genes were mainly concentrated in aminobenzoate degradation (A1), benzoate degradation (A2), lysine degradation (A3), and tryptophan metabolism (A4) pathways. The down-regulated genes were mainly concentrated aminoacyl-tRNA biosynthesis (B1), oxidative phosphorylation (B2), citrate cycle (B3), and peptidoglycan biosynthesis (B4) pathways.
(PDF)

Table S1 **The complete list of differentially expressed gene candidates in intracellular** ***Brucella abortus*** **by microarray analysis.**
(PDF)

Table S2 **Genes selected and primers used by qRT-PCR.**
(PDF)
