# Supplementary material for: Microarray-Based Identification of Differentially Expressed Genes in Intracellular Brucella abortus within RAW264.7 Cells
Source: PLoS One. 2013 Aug 7;8(8):e67014. doi: 10.1371/journal.pone.0067014 (PMC3737221; doi:10.1371/journal.pone.0067014)
Supplement: Figure S1 — Biological pathway of differentially expressed genes. Yellow nodes are associated with down-regulated genes; orange nodes are associated with up-regulated or only whole dataset genes; green nodes have no significance in expression. The up-regulated genes were mainly concentrated in aminobenzoate degradation (A1), benzoate degradation (A2), lysine degradation (A3), and tryptophan metabolism (A4) pathways. The down-regulated genes were mainly concentrated aminoacyl-tRNA biosynthesis (B1), oxidative phosphorylation (B2), citrate cycle (B3), and peptidoglycan biosynthesis (B4) pathways. (PDF) [file pone.0067014.s001.pdf]

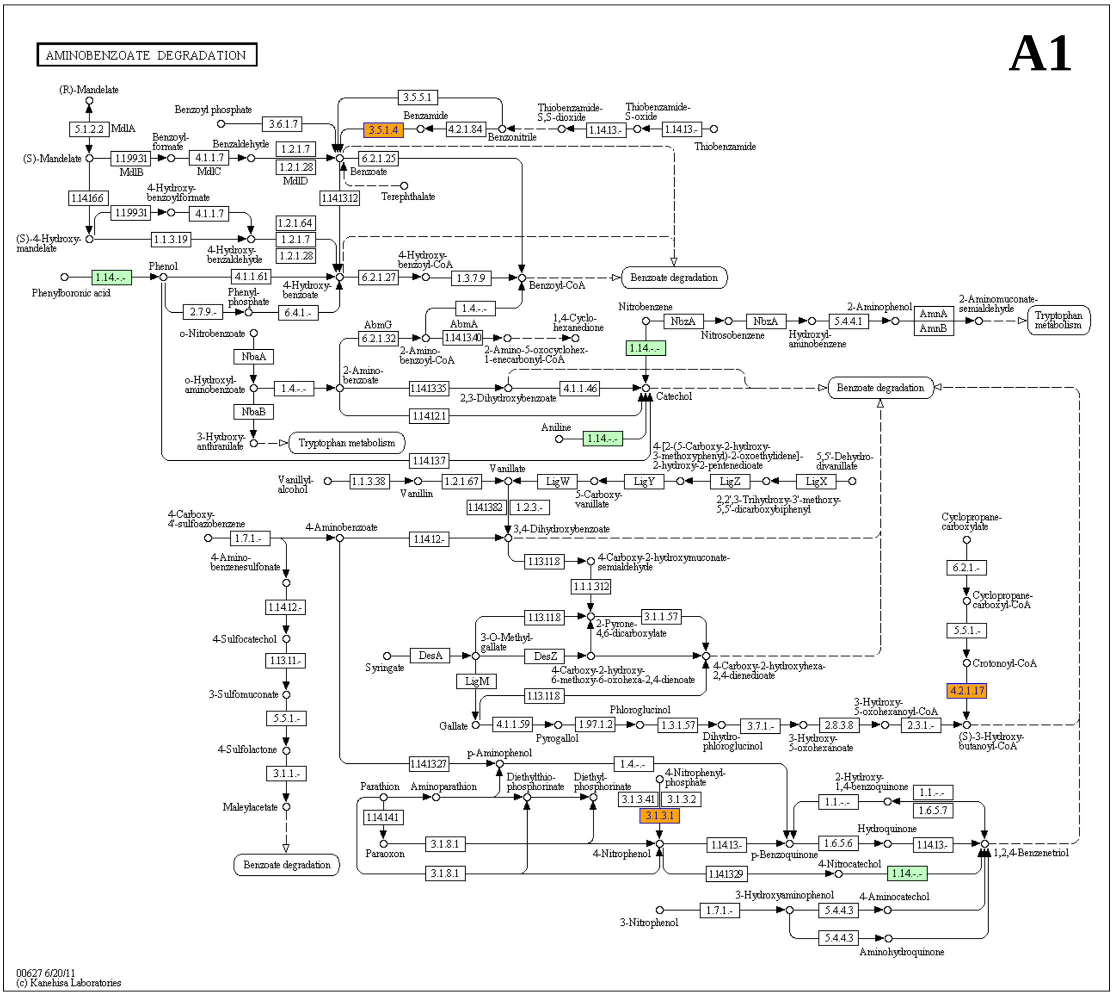
A1
AMINOBENZOATE DEGRADATION
(R)-Mandelate
(S)-Mandelate
(S)-4-Hydroxy-mandelate
Benzoyl phosphate
Benzoyl-formate
Benzaldehyde
4-Hydroxy-benzoylformate
4-Hydroxy-benzaldehyde
Benzamide
Benzonitrile
Thiobenzamide-S,S-dioxide
Thiobenzamide-S-oxide
Thiobenzamide
Benzoate
Terephthalate
Phenol
Phenylboronic acid
Phenyl-phosphate
4-Hydroxy-benzoate
4-Hydroxy-benzoyl-CoA
Benzoyl-CoA
Benzoate degradation
o-Nitrobenzoate
o-Hydroxyl-aminobenzoate
3-Hydroxy-anthranilate
Tryptophan metabolism
2-Amino-benzoyl-CoA
2-Amino-benzoate
2-Amino-5-oxocyclohex-1-enecarbonyl-CoA
1,4-Cyclo-hexanedione
2,3-Dihydroxybenzoate
Catechol
Aniline
Nitrobenzene
Nitrosobenzene
Hydroxyl-aminobenzene
2-Aminophenol
2-Aminomuconate-semialdehyde
Vanillyl-alcohol
Vanillin
Vanillate
5-Carboxy-vanillate
5,5'-Dehydro-divanillate
4-Carboxy-4'-sulfoazobenzene
4-Aminobenzoate
4-Amino-benzenesulfonate
3,4-Dihydroxybenzoate
4-Sulfocatechol
3-Sulfomuconate
4-Sulfolactone
Maleylacetate
Syringate
3-O-Methyl-gallate
2-Pyrone-4,6-dicarboxylate
4-Carboxy-2-hydroxyhexa-2,4-dienedioate
Gallate
Pyrogallol
Phloroglucinol
Dihydro-phloroglucinol
3-Hydroxy-5-oxohexanoate
3-Hydroxy-5-oxohexanoyl-CoA
(S)-3-Hydroxy-butanoyl-CoA
Cyclopropane-carboxylate
Cyclopropane-carboxyl-CoA
Crotonoyl-CoA
p-Aminophenol
Parathion
Aminoparathion
Paraoxon
4-Nitrophenol
4-Nitrophenyl-phosphate
p-Benzoquinone
2-Hydroxy-1,4-benzoquinone
Hydroquinone
4-Nitrocatechol
1,2,4-Benzenetriol
3-Hydroxyaminophenol
4-Aminocatechol
3-Nitrophenol
Aminohydroquinone
00627 6/20/11
(c) Kanehisa Laboratories

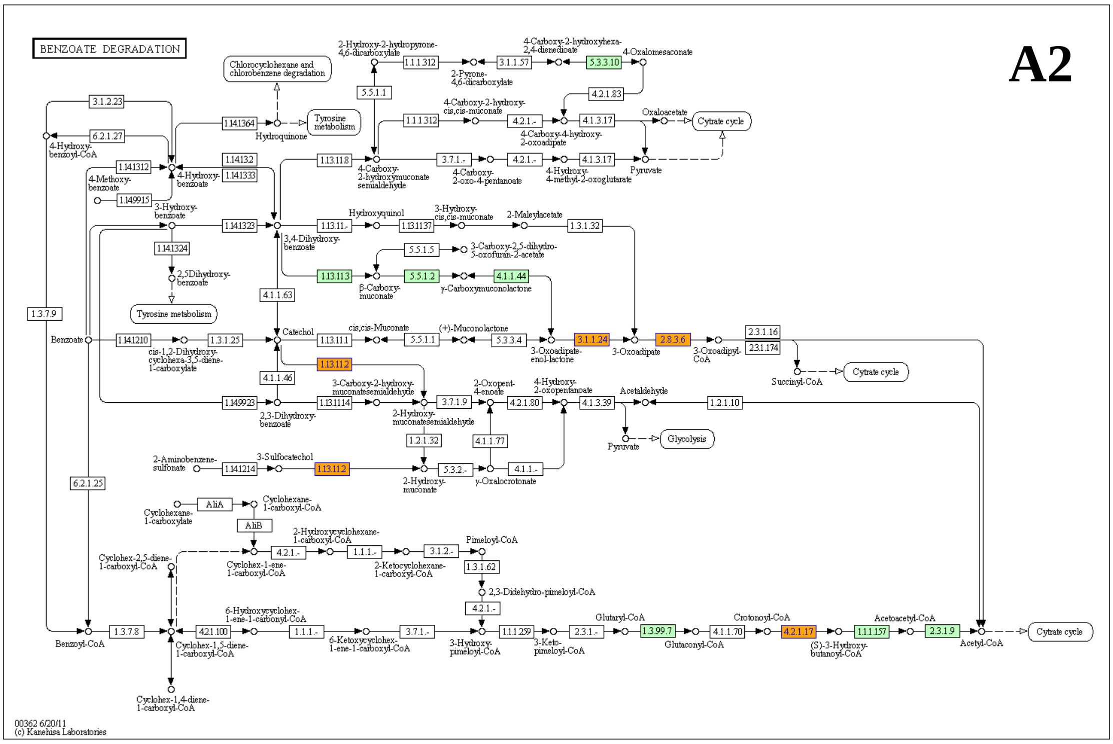
A2
BENZOATE DEGRADATION
Chlorocyclohexane and chlorobenzene degradation
Tyrosine metabolism
Hydroquinone
4-Hydroxy-benzoyl-CoA
4-Hydroxy-benzoate
4-Methoxy-benzoate
3-Hydroxy-benzoate
2,5-Dihydroxy-benzoate
3,4-Dihydroxy-benzoate
Catechol
Benzoate
cis-1,2-Dihydroxy-cyclohexa-3,5-diene-1-carboxylate
2,3-Dihydroxy-benzoate
2-Aminobenzene-sulfonate
3-Sulfocatechol
cis,cis-Muconate
(+)-Muconolactone
3-Oxoadipate-enol-lactone
3-Oxoadipate
3-Oxoadipyl-CoA
Succinyl-CoA
Citrate cycle
Oxaloacetate
Pyruvate
Acetaldehyde
Glycolysis
Cyclohexane-1-carboxylate
Cyclohexane-1-carboxyl-CoA
Cyclohex-1-ene-1-carboxyl-CoA
2-Ketocyclohexane-1-carboxyl-CoA
Pimeloyl-CoA
2,3-Didehydro-pimeloyl-CoA
3-Hydroxy-pimeloyl-CoA
3-Keto-pimeloyl-CoA
Glutaryl-CoA
Glutaconyl-CoA
Crotonoyl-CoA
(S)-3-Hydroxy-butanoyl-CoA
Acetoacetyl-CoA
Acetyl-CoA
Benzoyl-CoA
Cyclohex-1,5-diene-1-carboxyl-CoA
Cyclohex-1,4-diene-1-carboxyl-CoA
00362 6/20/11
(c) Kanehisa Laboratories

A3

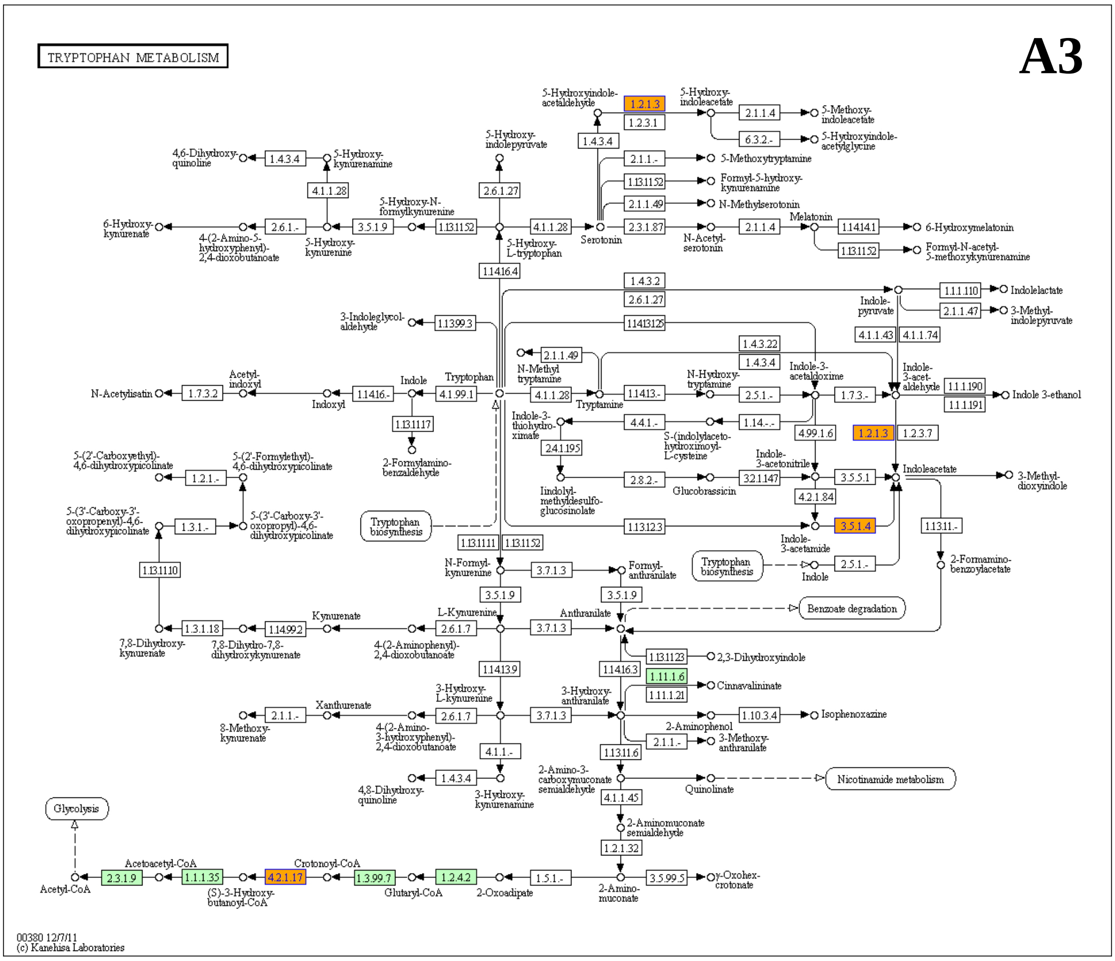
TRYPTOPHAN METABOLISM
A3
5-Hydroxyindole-acetaldehyde
1.2.1.3
1.2.3.1
5-Hydroxy-indoleacetate
2.1.1.4
5-Methoxy-indoleacetate
6.3.2.-
5-Hydroxyindole-acetylglycine
5-Hydroxy-indolepyruvate
1.4.3.4
2.1.1.-
5-Methoxytryptamine
1.13.11.52
Formyl-5-hydroxy-kynurenamine
2.1.1.49
N-Methylserotonin
4,6-Dihydroxy-quinoline
1.4.3.4
5-Hydroxy-kynurenamine
4.1.1.28
2.6.1.27
5-Hydroxy-N-formylkynurenine
6-Hydroxy-kynurenate
2.6.1.-
3.5.1.9
1.13.11.52
4.1.1.28
2.3.1.87
2.1.1.4
Melatonin
1.14.14.1
6-Hydroxymelatonin
4-(2-Amino-5-hydroxyphenyl)-2,4-dioxobutanoate
5-Hydroxy-kynurenine
5-Hydroxy-L-tryptophan
Serotonin
N-Acetyl-serotonin
1.13.11.52
Formyl-N-acetyl-5-methoxykynurenamine
1.14.16.4
1.4.3.2
2.6.1.27
Indole-pyruvate
1.1.1.110
Indolelactate
2.1.1.47
3-Methyl-indolepyruvate
3-Indoleglycol-aldehyde
1.13.99.3
1.14.13.125
4.1.1.43
4.1.1.74
2.1.1.49
1.4.3.22
N-Methyl tryptamine
1.4.3.4
Indole-3-acetaldoxime
Indole-3-acet-aldehyde
N-Acetylisatin
1.7.3.2
Acetyl-indoxyl
1.14.16.-
Indole
Tryptophan
4.1.99.1
4.1.1.28
1.14.13.-
N-Hydroxy-tryptamine
2.5.1.-
1.7.3.-
1.1.1.190
1.1.1.191
Indole 3-ethanol
Indoxyl
Tryptamine
1.13.11.17
Indole-3-thiohydro-ximate
4.4.1.-
1.14.-.-
4.99.1.6
1.2.1.3
1.2.3.7
2-Formylamino-benzaldehyde
2.4.1.195
S-(indolylaceto-hydroximoyl)-L-cysteine
Indole-3-acetonitrile
5-(2'-Carboxyethyl)-4,6-dihydroxypicolinate
1.2.1.-
5-(2'-Formylethyl)-4,6-dihydroxypicolinate
2.8.2.-
3.2.1.147
3.5.5.1
Indoleacetate
3-Methyl-dioxyindole
Indolyl-methylthiohydroximate
Glucobrassicin
4.2.1.84
5-(3'-Carboxy-3'-oxopropenyl)-4,6-dihydroxypicolinate
1.3.1.-
5-(3'-Carboxy-3'-oxopropyl)-4,6-dihydroxypicolinate
Tryptophan biosynthesis
1.13.12.3
3.5.1.4
1.13.11.-
Indole-3-acetamide
1.13.11.11
1.13.11.52
1.13.11.10
N-Formyl-kynurenine
3.7.1.3
Formyl-anthranilate
Tryptophan biosynthesis
2.5.1.-
Indole
2-Formamino-benzoylacetate
3.5.1.9
3.5.1.9
Benzoate degradation
7,8-Dihydroxy-kynurenate
1.3.1.18
1.14.99.2
Kynurenate
L-Kynurenine
Anthranilate
7,8-Dihydro-7,8-dihydroxykynurenate
4-(2-Aminophenyl)-2,4-dioxobutanoate
2.6.1.7
3.7.1.3
1.13.11.23
2,3-Dihydroxyindole
1.14.13.9
1.14.16.3
1.11.1.6
Cinnavalininate
1.11.1.21
3-Hydroxy-L-kynurenine
3-Hydroxy-anthranilate
Xanthurenate
2.1.1.-
2.6.1.7
3.7.1.3
1.10.3.4
Isophenoxazine
2-Aminophenol
8-Methoxy-kynurenate
4-(2-Amino-3-hydroxyphenyl)-2,4-dioxobutanoate
2.1.1.-
3-Methoxy-anthranilate
4.1.1.-
1.13.11.6
1.4.3.4
2-Amino-3-carboxymuconate semialdehyde
Quinolinate
Nicotinamide metabolism
4,8-Dihydroxy-quinoline
3-Hydroxy-kynurenamine
4.1.1.45
Glycolysis
2-Aminomuconate semialdehyde
1.2.1.32
Acetoacetyl-CoA
Crotonoyl-CoA
Acetyl-CoA
2.3.1.9
1.1.1.35
4.2.1.17
1.3.99.7
1.2.4.2
1.5.1.-
3.5.99.5
γ-Oxohex-crotonate
(S)-3-Hydroxy-butanoyl-CoA
Glutaryl-CoA
2-Oxoadipate
2-Amino-muconate
00380 12/7/11
(c) Kanehisa Laboratories

A4

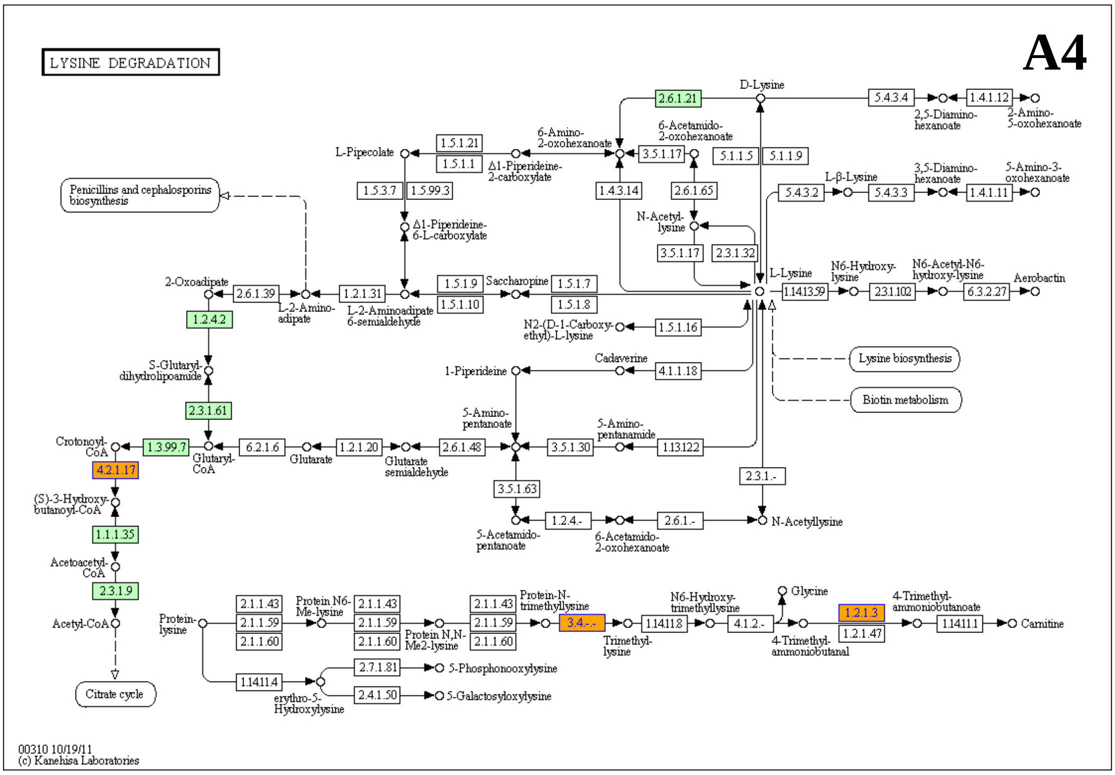
LYSINE DEGRADATION
A4
D-Lysine
2.6.1.21
5.4.3.4
2,5-Diamino-hexanoate
1.4.1.12
2-Amino-5-oxohexanoate
L-Pipecolate
1.5.1.21
1.5.1.1
6-Amino-2-oxohexanoate
Δ1-Piperideine-2-carboxylate
6-Acetamido-2-oxohexanoate
3.5.1.17
5.1.1.5
5.1.1.9
Penicillins and cephalosporins biosynthesis
1.5.3.7
1.5.99.3
1.4.3.14
2.6.1.65
L-β-Lysine
5.4.3.2
5.4.3.3
3,5-Diamino-hexanoate
1.4.1.11
5-Amino-3-oxohexanoate
Δ1-Piperideine-6-L-carboxylate
N-Acetyl-lysine
3.5.1.17
2.3.1.32
2-Oxoadipate
2.6.1.39
1.2.1.31
1.5.1.9
Saccharopine
1.5.1.7
L-Lysine
N6-Hydroxy-lysine
N6-Acetyl-N6-hydroxy-lysine
Aerobactin
1.14.13.59
2.3.1.102
6.3.2.27
L-2-Amino-adipate
L-2-Aminoadipate 6-semialdehyde
1.5.1.10
1.5.1.8
1.2.4.2
N2-(D-1-Carboxy-ethyl)-L-lysine
1.5.1.16
S-Glutaryl-dihydrolipoamide
Cadaverine
1-Piperideine
4.1.1.18
Lysine biosynthesis
Biotin metabolism
2.3.1.61
5-Amino-pentanoate
5-Amino-pentanamide
Crotonoyl-CoA
1.3.99.7
Glutaryl-CoA
6.2.1.6
Glutarate
1.2.1.20
Glutarate semialdehyde
2.6.1.48
3.5.1.30
1.13.12.2
4.2.1.17
3.5.1.63
2.3.1.-
(S)-3-Hydroxy-butanoyl-CoA
1.2.4.-
2.6.1.-
N-Acetyllysine
1.1.1.35
5-Acetamido-pentanoate
6-Acetamido-2-oxohexanoate
Acetoacetyl-CoA
2.3.1.9
Glycine
Protein N6-Me-lysine
Protein-N-trimethyllysine
N6-Hydroxy-trimethyllysine
4-Trimethyl-ammoniobutanoate
Acetyl-CoA
Protein-lysine
2.1.1.43
2.1.1.59
2.1.1.60
2.1.1.43
2.1.1.59
2.1.1.60
Protein N,N-Me2-lysine
2.1.1.43
2.1.1.59
2.1.1.60
3.4.-.-
1.14.11.8
4.1.2.-
1.2.1.3
1.2.1.47
1.14.11.1
Carnitine
Trimethyl-lysine
4-Trimethyl-ammoniobutanal
1.14.11.4
2.7.1.81
5-Phosphonooxylysine
erythro-5-Hydroxylysine
2.4.1.50
5-Galactosyloxylysine
Citrate cycle
00310 10/19/11
(c) Kanehisa Laboratories

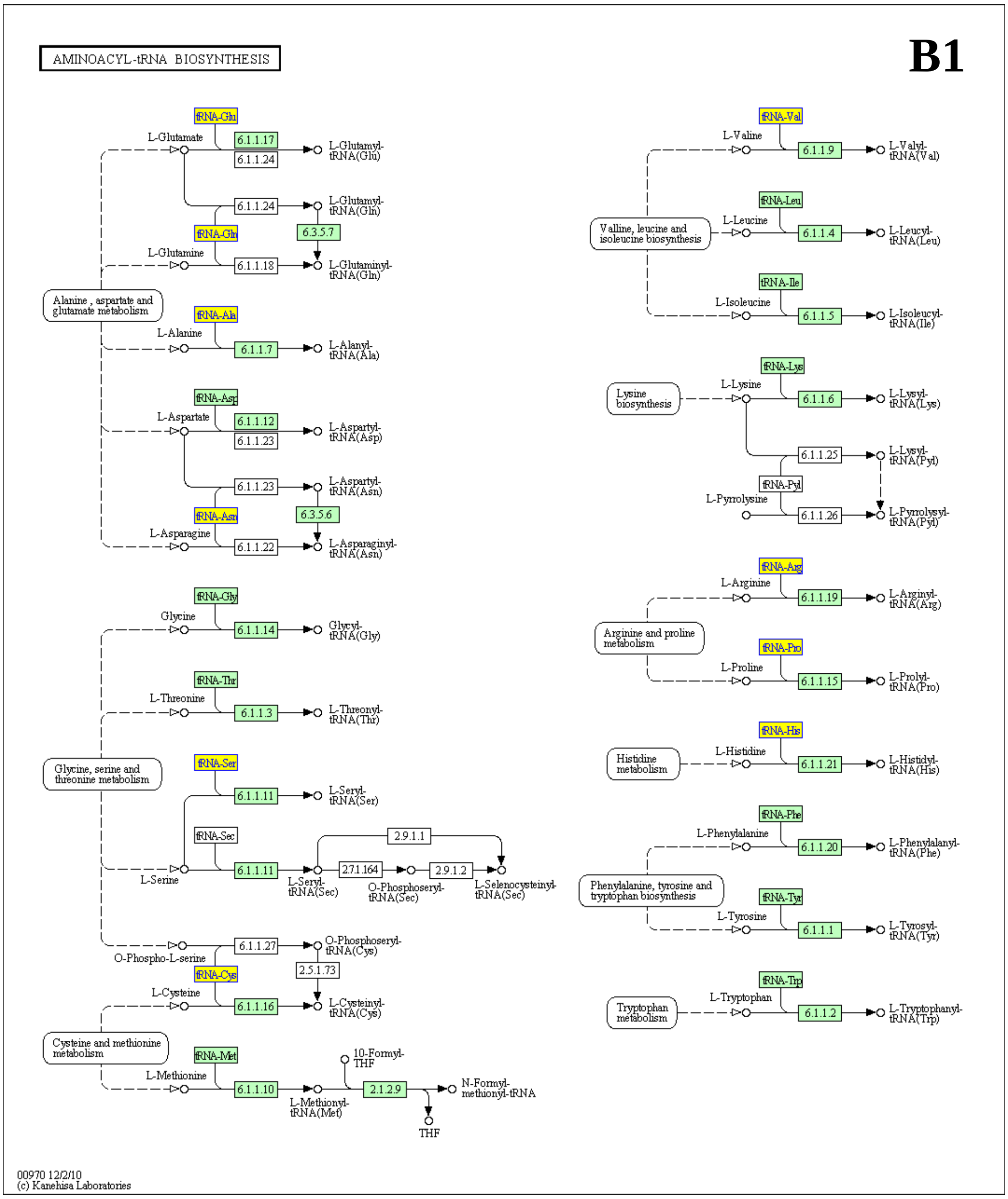
B1
AMINOACYL-tRNA BIOSYNTHESIS
tRNA-Glu
L-Glutamate
6.1.1.17
6.1.1.24
L-Glutamyl-tRNA(Glu)
6.1.1.24
L-Glutamyl-tRNA(Gln)
tRNA-Gln
6.3.5.7
L-Glutamine
6.1.1.18
L-Glutaminyl-tRNA(Gln)
Alanine, aspartate and glutamate metabolism
tRNA-Ala
L-Alanine
6.1.1.7
L-Alanyl-tRNA(Ala)
tRNA-Asp
L-Aspartate
6.1.1.12
6.1.1.23
L-Aspartyl-tRNA(Asp)
6.1.1.23
L-Aspartyl-tRNA(Asn)
tRNA-Asn
6.3.5.6
L-Asparagine
6.1.1.22
L-Asparaginyl-tRNA(Asn)
tRNA-Gly
Glycine
6.1.1.14
Glycyl-tRNA(Gly)
tRNA-Thr
L-Threonine
6.1.1.3
L-Threonyl-tRNA(Thr)
Glycine, serine and threonine metabolism
tRNA-Ser
6.1.1.11
L-Seryl-tRNA(Ser)
tRNA-Sec
2.9.1.1
6.1.1.11
2.7.1.164
2.9.1.2
L-Serine
L-Seryl-tRNA(Sec)
O-Phosphoseryl-tRNA(Sec)
L-Selenocysteinyl-tRNA(Sec)
6.1.1.27
O-Phosphoseryl-tRNA(Cys)
O-Phospho-L-serine
tRNA-Cys
2.5.1.73
L-Cysteine
6.1.1.16
L-Cysteinyl-tRNA(Cys)
Cysteine and methionine metabolism
tRNA-Met
10-Formyl-THF
L-Methionine
6.1.1.10
2.1.2.9
N-Formyl-methionyl-tRNA
L-Methionyl-tRNA(Met)
THF
tRNA-Val
L-Valine
6.1.1.9
L-Valyl-tRNA(Val)
tRNA-Leu
Valine, leucine and isoleucine biosynthesis
L-Leucine
6.1.1.4
L-Leucyl-tRNA(Leu)
tRNA-Ile
L-Isoleucine
6.1.1.5
L-Isoleucyl-tRNA(Ile)
tRNA-Lys
Lysine biosynthesis
L-Lysine
6.1.1.6
L-Lysyl-tRNA(Lys)
6.1.1.25
L-Lysyl-tRNA(Pyl)
tRNA-Pyl
L-Pyrrolysine
6.1.1.26
L-Pyrrolysyl-tRNA(Pyl)
tRNA-Arg
L-Arginine
6.1.1.19
L-Arginyl-tRNA(Arg)
Arginine and proline metabolism
tRNA-Pro
L-Proline
6.1.1.15
L-Prolyl-tRNA(Pro)
tRNA-His
Histidine metabolism
L-Histidine
6.1.1.21
L-Histidyl-tRNA(His)
tRNA-Phe
L-Phenylalanine
6.1.1.20
L-Phenylalanyl-tRNA(Phe)
Phenylalanine, tyrosine and tryptophan biosynthesis
tRNA-Tyr
L-Tyrosine
6.1.1.1
L-Tyrosyl-tRNA(Tyr)
tRNA-Trp
Tryptophan metabolism
L-Tryptophan
6.1.1.2
L-Tryptophanyl-tRNA(Trp)
00970 12/2/10
(c) Kanehisa Laboratories

B2

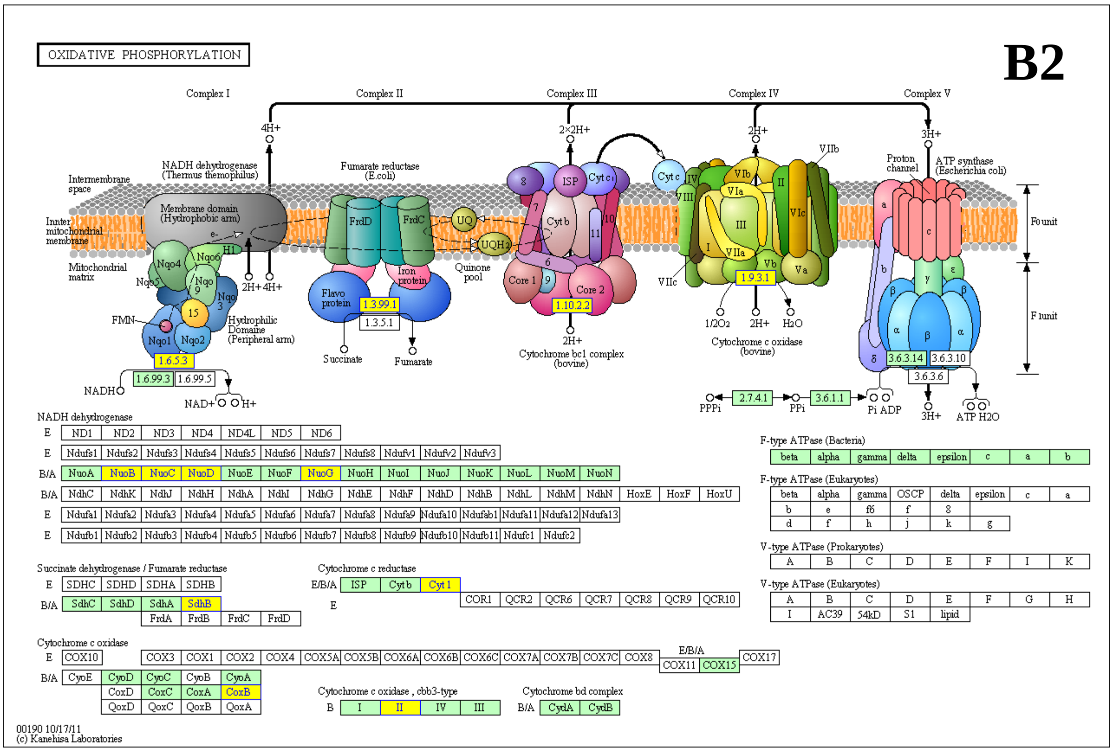
OXIDATIVE PHOSPHORYLATION
Complex I
Complex II
Complex III
Complex IV
Complex V
4H+
2×2H+
2H+
3H+
NADH dehydrogenase (Thermus thermophilus)
Fumarate reductase (E.coli)
Intermembrane space
Inner mitochondrial membrane
Mitochondrial matrix
Membrane domain (Hydrophobic arm)
Hydrophilic Domaine (Peripheral arm)
FMN
NADH
NAD+
H+
1.6.5.3
1.6.99.3
1.6.99.5
Flavo protein
Iron protein
Quinone pool
UQ
UQH2
1.3.99.1
1.3.5.1
Succinate
Fumarate
Cyt b
Cyt c
ISP
Core 1
Core 2
1.10.2.2
Cytochrome bc1 complex (bovine)
1.9.3.1
1/2O2
H2O
Cytochrome c oxidase (bovine)
Proton channel
ATP synthase (Escherichia coli)
F0 unit
F1 unit
3.6.3.14
3.6.3.10
3.6.3.6
2.7.4.1
3.6.1.1
PPPi
PPi
Pi ADP
ATP H2O
NADH dehydrogenase
E ND1 ND2 ND3 ND4 ND4L ND5 ND6
E Ndufs1 Ndufs2 Ndufs3 Ndufs4 Ndufs5 Ndufs6 Ndufs7 Ndufs8 Ndufv1 Ndufv2 Ndufv3
B/A NuoA NuoB NuoC NuoD NuoE NuoF NuoG NuoH NuoI NuoJ NuoK NuoL NuoM NuoN
B/A NdhC NdhK NdhJ NdhH NdhA NdhI NdhG NdhE NdhF NdhD NdhB NdhL NdhM NdhN HoxE HoxF HoxU
E Ndufa1 Ndufa2 Ndufa3 Ndufa4 Ndufa5 Ndufa6 Ndufa7 Ndufa8 Ndufa9 Ndufa10 Ndufab1 Ndufa11 Ndufa12 Ndufa13
E Ndufb1 Ndufb2 Ndufb3 Ndufb4 Ndufb5 Ndufb6 Ndufb7 Ndufb8 Ndufb9 Ndufb10 Ndufb11 Ndufc1 Ndufc2
Succinate dehydrogenase / Fumarate reductase
E SDHC SDHD SDHA SDHB
B/A SdhC SdhD SdhA SdhB
FrdA FrdB FrdC FrdD
Cytochrome c reductase
E/B/A ISP Cytb Cyt1
E COR1 QCR2 QCR6 QCR7 QCR8 QCR9 QCR10
Cytochrome c oxidase
E COX10 COX3 COX1 COX2 COX4 COX5A COX5B COX6A COX6B COX6C COX7A COX7B COX7C COX8
E/B/A COX11 COX15 COX17
B/A CyoE CyoD CyoC CyoB CyoA
CoxD CoxC CoxA CoxB
QoxD QoxC QoxB QoxA
Cytochrome c oxidase , cbb3-type
B I II IV III
Cytochrome bd complex
B/A CydA CydB
F-type ATPase (Bacteria)
beta alpha gamma delta epsilon c a b
F-type ATPase (Eukaryotes)
beta alpha gamma OSCP delta epsilon c a
b e f6 f 8
d f h j k g
V-type ATPase (Prokaryotes)
A B C D E F I K
V-type ATPase (Eukaryotes)
A B C D E F G H
I AC39 54kD S1 lipid
00190 10/17/11
(c) Kanehisa Laboratories

B3

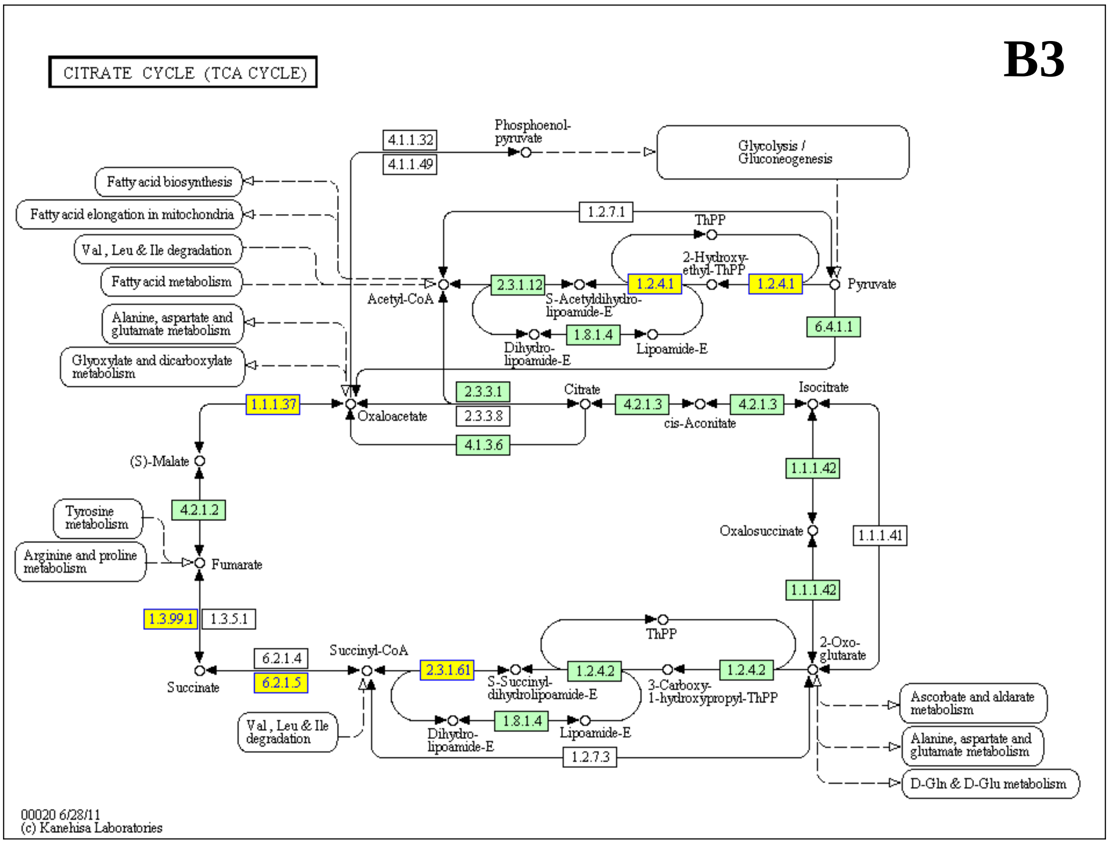
CITRATE CYCLE (TCA CYCLE)
4.1.1.32
4.1.1.49
Phosphoenol-pyruvate
Glycolysis / Gluconeogenesis
Fatty acid biosynthesis
Fatty acid elongation in mitochondria
Val , Leu & Ile degradation
Fatty acid metabolism
Alanine, aspartate and glutamate metabolism
Glyoxylate and dicarboxylate metabolism
1.2.7.1
ThPP
2-Hydroxy-ethyl-ThPP
2.3.1.12
1.2.4.1
1.2.4.1
Pyruvate
Acetyl-CoA
S-Acetyldihydro-lipoamide-E
Dihydro-lipoamide-E
1.8.1.4
Lipoamide-E
6.4.1.1
2.3.3.1
2.3.3.8
4.1.3.6
Citrate
4.2.1.3
4.2.1.3
cis-Aconitate
Isocitrate
1.1.1.37
Oxaloacetate
(S)-Malate
4.2.1.2
Tyrosine metabolism
Arginine and proline metabolism
Fumarate
1.3.99.1
1.3.5.1
1.1.1.42
Oxalosuccinate
1.1.1.41
1.1.1.42
2-Oxo-glutarate
6.2.1.4
6.2.1.5
Succinyl-CoA
Succinate
2.3.1.61
S-Succinyl-dihydrolipoamide-E
1.2.4.2
3-Carboxy-1-hydroxypropyl-ThPP
1.2.4.2
ThPP
Dihydro-lipoamide-E
1.8.1.4
Lipoamide-E
1.2.7.3
Val , Leu & Ile degradation
Ascorbate and aldarate metabolism
Alanine, aspartate and glutamate metabolism
D-Gln & D-Glu metabolism
00020 6/28/11
(c) Kanehisa Laboratories

B4

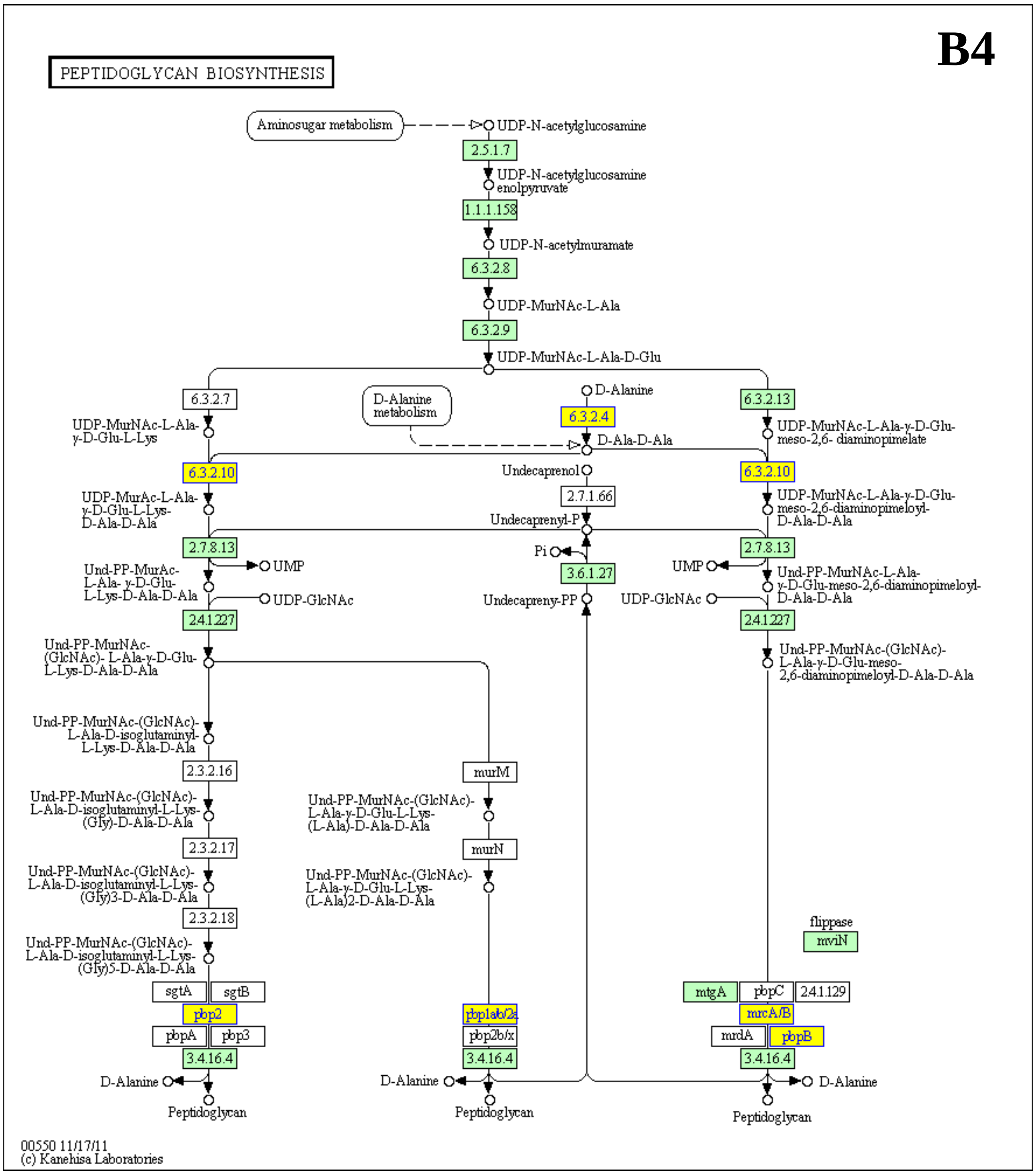

PEPTIDOGLYCAN BIOSYNTHESIS
Aminosugar metabolism
UDP-N-acetylglucosamine
2.5.1.7
UDP-N-acetylglucosamine enolpyruvate
1.1.1.158
UDP-N-acetylmuramate
6.3.2.8
UDP-MurNAc-L-Ala
6.3.2.9
UDP-MurNAc-L-Ala-D-Glu
6.3.2.7
D-Alanine metabolism
D-Alanine
6.3.2.4
6.3.2.13
UDP-MurNAc-L-Ala-γ-D-Glu-L-Lys
D-Ala-D-Ala
UDP-MurNAc-L-Ala-γ-D-Glu-meso-2,6- diaminopimelate
6.3.2.10
Undecaprenol
6.3.2.10
2.7.1.66
UDP-MurNAc-L-Ala-γ-D-Glu-L-Lys-D-Ala-D-Ala
UDP-MurNAc-L-Ala-γ-D-Glu-meso-2,6-diaminopimeloyl-D-Ala-D-Ala
Undecaprenyl-P
2.7.8.13
2.7.8.13
Pi
UMP
UMP
3.6.1.27
Und-PP-MurAc-L-Ala- γ-D-Glu-L-Lys-D-Ala-D-Ala
Und-PP-MurNAc-L-Ala-γ-D-Glu-meso-2,6-diaminopimeloyl-D-Ala-D-Ala
UDP-GlcNAc
Undecapreny-PP
UDP-GlcNAc
2.4.1.227
2.4.1.227
Und-PP-MurNAc-(GlcNAc)- L-Ala-γ-D-Glu-L-Lys-D-Ala-D-Ala
Und-PP-MurNAc-(GlcNAc)-L-Ala-γ-D-Glu-meso-2,6-diaminopimeloyl-D-Ala-D-Ala
Und-PP-MurNAc-(GlcNAc)-L-Ala-D-isoglutaminyl-L-Lys-D-Ala-D-Ala
2.3.2.16
murM
Und-PP-MurNAc-(GlcNAc)-L-Ala-D-isoglutaminyl-L-Lys-(Gly)-D-Ala-D-Ala
Und-PP-MurNAc-(GlcNAc)-L-Ala-γ-D-Glu-L-Lys-(L-Ala)-D-Ala-D-Ala
2.3.2.17
murN
Und-PP-MurNAc-(GlcNAc)-L-Ala-D-isoglutaminyl-L-Lys-(Gly)3-D-Ala-D-Ala
Und-PP-MurNAc-(GlcNAc)-L-Ala-γ-D-Glu-L-Lys-(L-Ala)2-D-Ala-D-Ala
2.3.2.18
flippase
mviN
Und-PP-MurNAc-(GlcNAc)-L-Ala-D-isoglutaminyl-L-Lys-(Gly)5-D-Ala-D-Ala
sgtA
sgtB
mtgA
pbpC
2.4.1.129
pbp2
pbp1a/b/2a
mrcA/B
pbpA
pbp3
pbp2b/x
mrdA
pbpB
3.4.16.4
3.4.16.4
3.4.16.4
D-Alanine
D-Alanine
D-Alanine
Peptidoglycan
Peptidoglycan
Peptidoglycan
00550 11/17/11
(c) Kanehisa Laboratories
