# Supplementary material for: Microarray-Based Identification of Differentially Expressed Genes in Intracellular Brucella abortus within RAW264.7 Cells
Source: PLoS One. 2013 Aug 7;8(8):e67014. doi: 10.1371/journal.pone.0067014 (PMC3737221; doi:10.1371/journal.pone.0067014)
Supplement: Table S1 — The complete list of differentially expressed gene candidates in intracellular Brucella abortus by microarray analysis. (PDF) [file pone.0067014.s002.pdf]

Table S1 The complete list of candidate differentially expressed genes of intracellular *Brucella abortus* in RAW264.7 by microarray analysis

Gene locus tag of *B . abortus* 9-941	Fold Change	Regulation	Gene	Product
BruAb1_2100	2.13	up	*dnaK*	molecular chaperone DnaK
BruAb2_0372	2.69	up	*rbsC-3*	ribose ABC transporter, permease protein
BruAb1_0783	2.24	up	*ribD*	RibD, riboflavin biosynthesis protein RibD
BruAb1_1084	2.11	up		hypothetical protein
BruAb2_0752	2.37	up		MarR family transcriptional regulator
BruAb1_0937	2.23	up	*qacH*	QacH, quaternary ammonium compound-resistance protein
BruAb1_1735	4.42	up		hypothetical protein
BruAb1_1075	3.47	up		hypothetical protein
BruAb1_0415	2.83	up		hypothetical protein
BruAb2_1096	12.82	up		homoprotocatechuate 2,3-dioxygenase
BruAb1_0736	2.60	up		hypothetical protein
BruAb1_1774	2.55	up		D-amino acid dehydrogenase small subunit
BruAb2_1092	2.15	up		aldehyde dehydrogenase family protein
BruAb1_0567	2.09	up		hypothetical protein
BruAb1_0744	2.06	up		hypothetical protein
BruAb2_0465	2.38	up		hypothetical protein
BruAb1_0380	2.21	up		guanine deaminase
BruAb2_0731	2.73	up	*bioF*	8-amino-7-oxononanoate synthase
BruAb1_0778	3.89	up		hypothetical protein
BruAb2_0123	4.71	up		
BruAb2_0860	3.86	up	*minC*	septum formation inhibitor
BruAb2_0501	2.20	up		transcriptional regulator
BruAb2_0189	4.18	up		hypothetical protein
BruAb1_1653	2.02	up	*exbB*	ExbB, biopolymer transport protein ExbB
BruAb1_2157	2.05	up		enoyl-CoA hydratase
BruAb2_0719	2.23	up		putative monovalent cation/H+ antiporter subunit E
BruAb1_trna_0003	5.61	up		tRNA-Phe
BruAb1_1442	8.54	up		glycosy hydrolase family protein
BruAb1_0018	2.18	up		biotin carboxylase
BruAb1_1381	8.35	up		pyridoxine 5'-phosphate synthase
BruAb2_0124	2.00	up	*motA*	flagellar motor protein MotA
BruAb2_1003	2.63	up	*prfC*	PrfC, peptide chain release factor 3
BruAb2_0028	2.24	up		branched-chain amino acid ABC transporter, ATP-binding protein
BruAb1_0083	2.29	up		lipase/acylhydrolase domain-containing protein
BruAb2_0572	5.01	up		renal dipeptidase family protein
BruAb1_0475	9.38	up		hypothetical protein
BruAb1_0292	12.63	up		hypothetical protein
BruAb1_0991	2.63	up		hypothetical protein
BruAb1_0667	2.25	up		hypothetical protein
BruAb2_0139	2.03	up		phthalate transporter

BruAb2_0634	3.22	up		hypothetical protein
BruAb2_0733	2.91	up	*bioA*	adenosylmethionine--8-amino-7-oxononanoate transaminase
BruAb1_0417	2.87	up		hypothetical protein
BruAb2_0869	3.05	up		hypothetical protein
BruAb1_0602	4.18	up		hypothetical protein
BruAb1_1183	3.43	up		hypothetical protein
BruAb1_0511	2.09	up		HAD superfamily hydrolase
BruAb1_1063	2.90	up		
BruAb2_0799	2.14	up		oxidoreductase
BruAb2_1108	2.06	up		hypothetical protein
BruAb2_0407	3.24	up		hypothetical protein
BruAb1_1931	2.05	up		amino acid ABC transporter, permease protein
BruAb1_0053	2.35	up		hypothetical protein
BruAb1_0416	3.00	up		hypothetical protein
BruAb2_0402	2.10	up		2-nitropropane dioxygenase family protein
BruAb2_0270	4.38	up		glyoxalase family protein
BruAb2_1009	10.23	up	*msrA*	methionine sulfoxide reductase A
BruAb1_0673	2.23	up	*fsr*	Fsr, fosmidomycin resistance protein
BruAb2_0204	4.17	up		
BruAb1_1558	2.26	up	*trpE*	anthranilate synthase
BruAb1_1246	2.16	up		hypothetical protein
BruAb1_trna_0002	2.51	up		tRNA-Pro
BruAb1_0316	2.71	up		TetR family transcriptional regulator
BruAb1_0505	6.55	up		processing protease
BruAb1_0212	5.07	up		D-amino acid oxidase family protein
BruAb1_1302	3.32	up		precorrin-3B C17-methyltransferase
BruAb1_1721	2.98	up		hypothetical protein
BruAb1_0806	2.18	up		hypothetical protein
BruAb2_0900	2.39	up	*nosX*	NosX
BruAb2_0298	2.82	up		sugar ABC transporter, permease protein
BruAb2_0358	3.07	up		hypothetical protein
BruAb1_0207	2.23	up		transcriptional regulator TenA
BruAb2_0242	2.19	up		hypothetical protein
BruAb1_1655	2.09	up		TonB-dependent receptor
BruAb2_0732	2.44	up	*bioD*	dithiobiotin synthetase
BruAb1_0625	2.12	up		outer membrane lipoprotein-related protein
BruAb2_0069	2.13	up		type IV secretion system protein VirB1
BruAb1_1068	16.52	up		hypothetical protein
BruAb1_1930	2.16	up		
BruAb2_1011	3.32	up		hypothetical protein
BruAb2_0075	2.02	up		SPFH domain-containing protein/band 7 family protein
BruAb1_2148	6.85	up		YaeC family lipoprotein

BruAb1_0636	2.19	up	*pepN*	aminopeptidase N
BruAb1_1717	2.12	up		hypothetical protein
BruAb1_0898	2.49	up	*surE*	stationary phase survival protein SurE
BruAb1_0035	2.41	up		hypothetical protein
BruAb1_1669	2.15	up		acetyltransferase
BruAb2_0365	6.26	up	*eryD*	erythritol transcriptional regulator
BruAb2_0447	2.77	up		peptide ABC transporter, permease protein
BruAb2_0920	2.88	up		hypothetical protein
BruAb2_1050	2.31	up		
BruAb1_2005	2.42	up		hypothetical protein
BruAb1_1562	3.13	up		hypothetical protein
BruAb1_trna_0023	4.27	up		tRNA-Tyr
BruAb1_0795	2.25	up	*sugE*	SugE, SugE protein
BruAb1_0684	4.35	up		aminomethyltransferase
BruAb1_0231	2.90	up		sugar ABC transporter, permease protein
BruAb1_trna_0028	2.43	up		tRNA-Met
BruAb2_0546	3.96	up		amino acid ABC transporter, permease protein
BruAb2_0931	2.62	up	*norC*	NorC, nitric-oxide reductase, small subunit
BruAb2_1099	2.59	up		MarR family transcriptional regulator
BruAb2_0369	2.14	up		hypothetical protein
BruAb2_0734	2.46	up		3-oxoacyl-(acyl carrier protein) synthase II
BruAb2_0928	3.32	up	*norD*	NorD protein
BruAb2_0638	2.17	up		hypothetical protein
BruAb2_0772	5.89	up		
BruAb1_0414	2.37	up		hypothetical protein
BruAb1_0858	2.18	up		hypothetical protein
BruAb1_1301	2.16	up	*cobM*	CobM, precorrin-4 C11-methyltransferase
BruAb2_0599	2.62	up		hypothetical protein
BruAb1_0013	2.49	up		hypothetical protein
BruAb2_0592	2.22	up		
BruAb2_0957	4.01	up		transporter
BruAb1_1305	12.68	up		hypothetical protein
BruAb1_2032	2.53	up	*holA*	DNA polymerase III subunit delta
BruAb1_0045	2.08	up	*qoxD*	QoxD, ubiquinol oxidase subunit IV
BruAb2_0263	2.12	up		oxidoreductase
BruAb1_1092	3.48	up		LysR family transcriptional regulator
BruAb1_0603	5.15	up		hypothetical protein
BruAb2_1072	2.86	up	*flbT*	flagellar biosynthesis repressor FlbT
BruAb2_0837	2.16	up		EAL domain-containing protein
BruAb1_0638	2.69	up		transporter
BruAb1_1730	2.20	up	*purK*	phosphoribosylaminoimidazole carboxylase ATPase subunit
BruAb2_0583	2.50	up	*pcaL*	PcaL, 3-oxoadipate enol-lactone hydrolase

BruAb1_0976	2.85	up		
BruAb1_0752	2.48	up		hypothetical protein
BruAb2_0430	15.92	up	*nikC*	nickel transporter permease NikC
BruAb1_0188	2.20	up	*cysD*	sulfate adenylyltransferase subunit 2
BruAb2_trna_0006	7.07	up		tRNA-Lys
BruAb1_2031	2.82	up		ABC transporter, ATP-binding protein
BruAb1_1542	2.42	up		polyA polymerase family protein
BruAb1_0004	2.86	up	*moeB*	molybdopterin biosynthesis protein MoeB
BruAb2_0089	2.18	up	*modB*	molybdate ABC transporter permease protein
BruAb1_1485	3.44	up		MutT/nudix family protein
BruAb2_0015	2.93	up	*entE*	enterobactin synthase subunit E
BruAb1_1662	2.55	up		hypothetical protein
BruAb1_0702	17.45	up	*pdxA*	4-hydroxythreonine-4-phosphate dehydrogenase
BruAb1_0052	5.96	up		hypothetical protein
BruAb2_0281	3.76	up		LysR family transcriptional regulator
BruAb2_0014	4.52	up	*entB*	EntB, isochorismatase
BruAb1_0932	2.34	up		hypothetical protein
BruAb1_1360	2.07	up		urea transporter
BruAb1_1874	2.08	up	*tesB*	TesB, acyl-CoA thioesterase II
BruAb1_0655	2.08	up	*omp2a*	Omp2a, porin
BruAb2_1042	2.41	up		amidase
BruAb1_0933	2.72	up	*bcp*	Bcp, bacterioferritin comigratory protein
BruAb2_0240	2.42	up	*rbsC-5*	ribose ABC transporter, permease protein
BruAb1_1507	2.32	up		hypothetical protein
BruAb2_0428	2.24	up		
BruAb1_1187	4.06	up		hypothetical protein
BruAb2_0968	2.05	up	*dapD*	2,3,4,5-tetrahydropyridine-2,6-carboxylate N-succinyltransferase
BruAb2_0730	2.91	up	*bioB*	BioB, biotin synthase
BruAb1_1574	2.01	up		hypothetical protein
BruAb2_0363	4.20	up	*tpiA*	triosephosphate isomerase
BruAb1_0922	2.09	up		aminotransferase, class I
BruAb1_0016	9.08	up		enoyl-CoA hydratase
BruAb1_1597	2.52	up		
BruAb1_0044	2.28	up		ubiquinol oxidase subunit III
BruAb2_0277	13.92	up		branched-chain amino acid ABC transporter, permease protein
BruAb2_1128	10.01	up		AraC family transcriptional regulator
BruAb2_0375	2.24	up		hypothetical protein
BruAb1_1955	3.58	up		hypothetical protein
BruAb1_0271	2.10	up		hypothetical protein
BruAb1_1265	2.34	up	*cysE*	CysE, serine acetyltransferase
BruAb1_0905	2.28	up		hypothetical protein
BruAb1_0656	2.29	up		hypothetical protein

BruAb2_0119	2.08	up	*flhB*	flagellar biosynthesis protein FlhB
BruAb1_1452	3.47	up		hypothetical protein
BruAb1_1619	2.11	up	*rbsC-2*	RbsC-2, ribose ABC transporter, permease protein
BruAb2_0346	2.30	up		MarR family transcriptional regulator
BruAb1_1760	3.32	up	*ispA*	IspA, geranyltranstransferase
BruAb1_1490	2.52	up		hypothetical protein
BruAb2_0938	2.17	up		hypothetical protein
BruAb1_1962	2.49	up		hypothetical protein
BruAb1_1272	3.32	up	*recJ*	RecJ, single-stranded-DNA-specific exonuclease RecJ
BruAb1_1936	2.10	up		L-asparaginase type II
BruAb1_1738	2.04	up		ABC transporter, ATP-binding protein
BruAb2_0932	4.49	up	*norF*	norF protein
BruAb1_0495	3.02	up		
BruAb1_1217	3.05	up	*adk*	adenylate kinase
BruAb2_0063	2.79	up		type IV secretion system protein VirB7
BruAb2_0807	2.08	up		branched-chain amino acid ABC transporter, ATP-binding protein
BruAb2_0530	2.11	up		iron compound ABC transporter periplasmic iron compound-binding protein
BruAb2_0310	2.35	up	*alr*	Alr, alanine racemase
BruAb1_1032	6.91	up		
BruAb1_0980	2.08	up		hypothetical protein
BruAb1_0566	2.59	up	*rbsB-1*	RbsB-1, ribose ABC transporter
BruAb2_0588	3.23	up	*pcaI*	PcaI, 3-oxoadipate CoA-transferase, alpha subunit
BruAb2_0692	16.13	up		major facilitator family transporter
BruAb2_0457	2.08	up		hypothetical protein
BruAb1_1837	4.57	up		hypothetical protein
BruAb1_trna_0017	2.94	up		tRNA-Gly
BruAb2_0628	2.01	up		metal-dependent hydrolase
BruAb2_0058	2.15	up		lipoprotein
BruAb1_1972	3.74	up	*ftsE*	FtsE, cell division ABC transporter, ATP-binding protein
BruAb1_0015	2.49	up		hypothetical protein
BruAb1_0086	2.85	up		hypothetical protein
BruAb2_0143	2.07	up		hydroxypyruvate isomerase
BruAb2_1081	3.86	up	*motB*	flagellar motor protein MotB
BruAb1_trna_0012	9.33	up		tRNA-Asp
BruAb2_0994	2.08	up		nitroreductase family protein
BruAb2_0391	3.17	up	*hsdM*	HsdM, type I restriction-modification system, M subunit
BruAb2_0259	2.53	up		hypothetical protein
BruAb2_0806	2.04	up		branched-chain amino acid ABC transporter, ATP-binding protein
BruAb1_2028	2.54	up		ABC transporter periplasmic substrate-binding protein
BruAb1_0593	7.78	up		hypothetical protein
BruAb2_0321	2.00	up	*nagB*	NagB, glucosamine-fructose-6-phosphate aminotransferase
BruAb1_trna_0022	5.29	up		tRNA-Gly

BruAb2_0720	3.03	up		putative monovalent cation/H+ antiporter subunit D
BruAb1_1049	8.33	up		hypothetical protein
BruAb2_0405	2.01	up		hypothetical protein
BruAb2_0017	2.73	up		enterobactin synthetase subunit F
BruAb1_0284	2.45	up		phage integrase family site specific recombinase
BruAb2_0986	2.13	up		hypothetical protein
BruAb2_0126	8.76	up	*flgF*	flagellar basal body rod protein FlgF
BruAb1_2124	45.46	up		protease
BruAb1_0458	3.89	up	*dxs*	1-deoxy-D-xylulose-5-phosphate synthase
BruAb1_1900	2.04	up		hypothetical protein
BruAb1_0205	6.94	up		aldehyde dehydrogenase family protein
BruAb1_1444	3.22	up		transporter
BruAb2_0422	2.48	up		ABC transporter, periplasmic substrate-binding protein
BruAb2_0844	2.24	up		hypothetical protein
BruAb1_2027	2.44	up		hypothetical protein
BruAb1_trna_0026	3.90	up		tRNA-Lys
BruAb2_0753	6.23	up		ABC transporter, periplasmic substrate-binding protein
BruAb2_0663	2.90	up		hypothetical protein
BruAb2_0400	2.62	up		hypothetical protein
BruAb1_1926	8.36	up		hypothetical protein
BruAb2_0640	2.72	up	*ccdA*	CcdA, cytochrome c-type biogenesis protein CdcA
BruAb1_1814	7.61	up		hypothetical protein
BruAb1_1873	2.11	up	*amt*	Amt, ammonium transporter
BruAb2_0709	2.28	up		GntR family transcriptional regulator
BruAb1_1553	2.08	up		hypothetical protein
BruAb1_0973	2.14	up		hypothetical protein
BruAb1_1989	4.34	up		hypothetical protein
BruAb1_0043	2.03	up	*qoxB*	
BruAb1_0699	2.26	up	*rarD*	RarD, RarD protein
BruAb1_1085	2.02	up		hypothetical protein
BruAb1_1205	13.30	up	*phoA*	alkaline phosphatase
BruAb2_0750	3.59	up		HlyD family secretion protein
BruAb2_0919	2.05	up		copper-containing nitrite reductase
BruAb1_0291	7.13	up		hypothetical protein
BruAb1_1957	2.07	up	*argH*	argininosuccinate lyase
BruAb1_0427	5.27	up	*glyS*	glycyl-tRNA synthetase subunit beta
BruAb1_2132	2.81	down		Cro/CI family transcriptional regulator
BruAb1_0704	2.75	down		organic solvent tolerance protein
BruAb1_1276	2.24	down		aminotransferase
BruAb2_0636	2.21	down		sensory box protein
BruAb1_0339	2.01	down		hypothetical protein
BruAb2_0201	2.50	down		RNA pseudouridylate synthase family protein

BruAb2_0267	2.55	down	*rpsU*	30S ribosomal protein S21
BruAb1_0542	2.20	down	*rfbE*	RfbE, O-antigen export system ATP-binding protein RfbE
BruAb1_1551	5.32	down		hypothetical protein
BruAb2_0741	3.08	down		hypothetical protein
BruAb1_1239	2.32	down	*rpsJ*	30S ribosomal protein S10
BruAb1_0597	2.50	down		hypothetical protein
BruAb1_0772	2.35	down		arginyl-tRNA-protein transferase
BruAb1_1244	2.81	down		hypothetical protein
BruAb1_2008	2.34	down		lipoprotein
BruAb1_0817	2.04	down	*nuoB*	NADH dehydrogenase subunit B
BruAb1_1196	2.54	down		hypothetical protein
BruAb1_1728	2.26	down		hypothetical protein
BruAb1_0753	2.68	down		
BruAb1_2130	2.81	down		CBS domain-containing protein
BruAb1_0488	2.11	down		hypothetical protein
BruAb1_1530	2.19	down		ubiquinol-cytochrome c reductase, cytochrome c1
BruAb1_0330	2.24	down	*apaG*	ApaG
BruAb2_0043	3.10	down		response regulator
BruAb1_1877	2.31	down	*sdhB*	succinate dehydrogenase iron-sulfur subunit
BruAb1_1901	2.61	down	*sucD*	succinyl-CoA synthetase subunit alpha
BruAb1_1737	2.76	down		hypothetical protein
BruAb1_1883	2.15	down	*rplS*	50S ribosomal protein L19
BruAb2_0989	2.23	down	*glk*	glucokinase
BruAb1_0723	2.20	down		hypothetical protein
BruAb2_0061	24.85	down		type IV secretion system protein VirB9
BruAb1_1648	2.61	down		hypothetical protein
BruAb1_trna_0024	4.71	down		tRNA-Glu
BruAb1_0822	2.76	down	*nuoG*	NADH dehydrogenase subunit G
BruAb1_0280	2.34	down	*hisD*	histidinol dehydrogenase
BruAb1_0489	3.43	down	*coxB*	CoxB, cytochrome c oxidase, subunit II
BruAb2_0699	5.53	down		2-oxoisovalerate dehydrogenase E1 component, beta subunit
BruAb1_trna_0025	2.72	down		tRNA-Glu
BruAb1_1851	3.55	down	*ubiG*	
BruAb1_0483	2.81	down	*fabF*	3-oxoacyl-(acyl carrier protein) synthase II
BruAb1_1159	2.25	down	*lpxD*	UDP-3-O-[3-hydroxymyristoyl] glucosamine N-acyltransferase
BruAb1_0199	4.22	down		hypothetical protein
BruAb1_trna_0029	5.28	down		tRNA-His
BruAb1_0197	3.28	down		
BruAb1_1446	3.70	down		ArsR family transcriptional regulator
BruAb1_1423	2.65	down	*ddl*	D-alanine--D-alanine ligase
BruAb1_0099	5.07	down		response regulator
BruAb2_0393	3.31	down		hypothetical protein

BruAb2_0613	4.51	down	*divK*	DivK, polar differentiation response regulator
BruAb1_1506	2.68	down		cold-shock family protein
BruAb2_trna_0004	20.94	down		tRNA-Asn
BruAb1_0900	2.07	down		M24/M37 family peptidase
BruAb1_0234	2.06	down		mandelate racemase/muconate lactonizing enzyme family protein
BruAb1_trna_0041	2.29	down		tRNA-Arg
BruAb1_trna_0040	2.99	down		tRNA-Ala
BruAb1_trna_0034	2.73	down		tRNA-Glu
BruAb2_0858	2.26	down	*minE*	cell division topological specificity factor MinE
BruAb1_1748	2.67	down	*rpoH2*	RNA polymerase factor sigma-32
BruAb1_1179	4.20	down		hypothetical protein
BruAb1_1310	2.28	down		hypothetical protein
BruAb2_0537	3.32	down		sugar ABC transporter, periplasmic sugar-binding protein
BruAb1_0440	2.89	down		hypothetical protein
BruAb2_0296	3.72	down		
BruAb1_0202	3.33	down		hypothetical protein
BruAb1_1079	2.13	down		NifU-related protein
BruAb1_1432	2.11	down		penicillin-binding protein
BruAb2_0295	3.03	down		2-dehydro-3-deoxy-6-phosphogalactonate aldolase
BruAb1_0170	2.04	down		
BruAb1_1445	4.78	down	*metF*	MetF, 5,10-methylenetetrahydrofolate reductase
BruAb1_1675	2.17	down	*glmM*	phosphoglucosamine mutase
BruAb2_0170	3.04	down		hypothetical protein
BruAb1_1751	2.45	down	*fdxA*	FdxA, ferredoxin A
BruAb1_1903	2.13	down	*mdh*	malate dehydrogenase
BruAb1_1686	2.30	down		hypothetical protein
BruAb1_0215	2.19	down		copper-translocating P-type ATPase
BruAb2_rrna_0002	3.59	down		23S ribosomal RNA
BruAb1_1209	2.14	down		OmpA family protein
BruAb1_1944	2.65	down		
BruAb1_1328	2.68	down	*cysW-2*	CysW-2, sulfate ABC transporter, permease protein
BruAb1_1789	2.19	down		hypothetical protein
BruAb1_0968	2.57	down		
BruAb1_0687	2.05	down		hypothetical protein
BruAb1_0598	2.63	down		sensor histidine kinase
BruAb2_0700	6.50	down		2-oxoisovalerate dehydrogenase E1 component, alpha subunit
BruAb1_1430	2.78	down	*murF*	MurF, UDP-N-acetylmuramoylalanyl-D-glutamyl-2, 6-diaminopimelate--D-alanyl-D-alanyl ligase
BruAb1_0970	3.10	down		hypothetical protein
BruAb1_0049	2.19	down		hypothetical protein
BruAb1_0892	2.07	down		hypothetical protein
BruAb1_0748	2.17	down		hypothetical protein
BruAb2_trna_0007	5.81	down		tRNA-Ser

BruAb1_trna_0007	3.17	down		tRNA-Ser
BruAb1_trna_0005	5.97	down		tRNA-Arg
BruAb1_0705	2.17	down		hypothetical protein
BruAb1_0388	2.52	down	*ccoO*	cbb3-type cytochrome c oxidase subunit II
BruAb2_0464	2.54	down		glutamine synthetase family protein
BruAb1_0818	2.29	down	*nuoC*	NADH dehydrogenase subunit C
BruAb2_0685	3.08	down		oligopeptide ABC transporter, periplasmic oligopeptide-binding protein
BruAb1_1433	2.55	down		hypothetical protein
BruAb1_trna_0015	3.42	down		tRNA-Val
BruAb1_1311	2.39	down		hypothetical protein
BruAb1_1736	2.64	down		hypothetical protein
BruAb1_0357	2.64	down	*fpr*	Fpr, ferredoxin--NADP reductase
BruAb1_1101	2.11	down	*coaD*	phosphopantetheine adenylyltransferase
BruAb2_trna_0005	6.37	down		tRNA-Cys
BruAb1_1102	2.02	down		hypothetical protein
BruAb1_0764	2.04	down		hypothetical protein
BruAb1_1626	2.09	down		hypothetical protein
BruAb1_1330	6.68	down		sulfate ABC transporter sulfate-binding protein
BruAb1_0766	2.31	down		Ppx/GppA family phosphatase
BruAb1_1910	2.33	down	*ftsY*	FtsY, signal recognition particle-docking protein
BruAb1_1427	2.03	down	*ftsW*	FtsW, cell division protein
BruAb2_0527	2.19	down	*sodC*	SodC, superoxide dismutase, Cu-Zn
BruAb2_0506	2.25	down	*gcvP*	glycine dehydrogenase
BruAb1_1816	3.26	down		carboxyl-terminal protease
BruAb1_1401	3.08	down		hypothetical protein
BruAb1_1502	2.53	down		long-chain acyl-CoA thioester hydrolase
BruAb1_1554	2.10	down		hypothetical protein
BruAb1_0679	2.56	down		hypothetical protein
BruAb1_0890	4.25	down		hypothetical protein
BruAb1_1188	2.52	down		hypothetical protein
BruAb1_rrna_0002	3.08	down		23S ribosomal RNA
BruAb1_2137	2.71	down		hypothetical protein
BruAb1_0579	2.03	down		death-on-curing family protein
BruAb1_trna_0010	11.56	down		tRNA-Pro
BruAb1_1538	2.90	down		hypothetical protein
BruAb1_2141	2.04	down	*rpsO*	30S ribosomal protein S15
BruAb1_1685	2.07	down	*tolQ*	TolQ, tolQ protein
BruAb1_trna_0016	5.62	down		tRNA-Arg
BruAb1_2070	2.80	down	*ptsH*	PtsH, phosphocarrier protein HPr
BruAb1_0703	2.13	down		peptidyl-prolyl cis-trans isomerase
BruAb1_1560	2.19	down		
BruAb2_0561	2.95	down		hypothetical protein

BruAb1_0949	2.92	down		
BruAb1_1135	2.04	down	*pdhA*	PdhA, pyruvate dehydrogenase complex, E1 component, alpha subunit
BruAb1_0156	3.21	down	*yfiA*	YfiA, ribosomal subunit interface protein
BruAb1_2086	2.55	down	*trpF*	N-(5'-phosphoribosyl)anthranilate isomerase
BruAb1_1740	2.20	down		lipoprotein
BruAb1_0754	2.62	down		lipoprotein
BruAb1_1374	2.88	down		aminotransferase, class I
BruAb2_0735	4.83	down		
BruAb1_2111	2.01	down		hypothetical protein
BruAb1_2155	3.14	down		pilus biosythesis protein-related protein
BruAb2_0606	3.93	down		hypothetical protein
BruAb1_1395	2.51	down		hypothetical protein
BruAb1_0078	2.16	down		hypothetical protein
BruAb1_1580	4.31	down		hypothetical protein
BruAb1_2066	2.17	down	*bvrS*	BvrS, sensor histidine kinase
BruAb1_1050	2.88	down		MutT/nudix family protein
BruAb1_trna_0006	25.37	down		tRNA-Gln
BruAb1_1644	3.04	down		two-component response regulator
BruAb1_0181	2.23	down	*ogt*	Ogt, methylated-DNA--protein-cysteine methyltransferase
BruAb2_0308	2.09	down		transcriptional regulator
BruAb1_1943	3.44	down		
BruAb1_1353	2.29	down	*ureA*	urease subunit gamma
BruAb1_1421	2.28	down	*ftsA*	FtsA, cell division protein FtsA
BruAb1_1898	2.40	down	*sucB*	dihydrolipoamide succinyltransferase
BruAb1_1820	2.74	down		iojap-related protein
BruAb1_0648	2.28	down		hypothetical protein
BruAb2_0922	2.08	down		hypothetical protein
BruAb2_0265	2.66	down		nodulation protein N
BruAb2_0811	2.84	down		hypothetical protein
BruAb1_0604	2.46	down		penicillin-binding protein
BruAb1_0341	2.68	down		LuxR family DNA-binding response regulator
BruAb1_2069	3.03	down		PTS system, IIA component
BruAb2_0078	2.33	down		homospermidine synthase
BruAb1_1255	2.13	down	*tuf*	elongation factor Tu
BruAb2_0603	2.01	down		hypothetical protein
BruAb2_0325	3.95	down		aldehyde dehydrogenase family protein
BruAb1_0657	2.20	down	*omp2b*	Omp2b, porin
BruAb1_1914	3.36	down		hypothetical protein
BruAb1_0727	2.16	down		hypothetical protein
BruAb1_0993	27.39	down		hypothetical protein
BruAb1_0819	3.09	down	*nuoD*	NADH dehydrogenase subunit D
BruAb1_0599	3.26	down		peptidoglycan-binding protein

BruAb1_1317	2.18	down		hypothetical protein
BruAb1_1178	2.54	down		D-alanyl-D-alanine carboxypeptidase
BruAb1_1240	2.56	down	*tuf*	elongation factor Tu
BruAb2_0213	2.35	down		hypothetical protein
BruAb1_trna_0032	3.46	down		tRNA-Ala
BruAb2_0459	2.20	down		AsnC family transcriptional regulator
