# Supplementary material for: Microarray-Based Identification of Differentially Expressed Genes in Intracellular Brucella abortus within RAW264.7 Cells
Source: PLoS One. 2013 Aug 7;8(8):e67014. doi: 10.1371/journal.pone.0067014 (PMC3737221; doi:10.1371/journal.pone.0067014)
Supplement: Table S2 — Genes selected and primers used by qRT-PCR. (PDF) [file pone.0067014.s003.pdf]

Table S2. Genes selected and primers used by qRT-PCR

Gene Name	Forward primer sequences (5'-3')	Reverse primer sequences (5'-3')	Products (bp)
BruAb2_1096	CGCTCTATAAGGGCGTGAAG	TGCCATTGGTGAAGGTGATA	198
BruAb1_0292	AGGGTTCAACACGGTCGTAG	GATCAGTTCCCGGTCTTTCA	103
BruAb1_0702	CACACCGGTTATGATGCTTG	ACGATTTCCGTCGTGGTAAG	105
BruAb1_1068	ATTCTGGTGGATCAGCTTGG	GCCCAGATCAAGCATACGTT	102
BruAb1_1205	ACAGCCAATCAGGACAAACC	AGTGCGCTTCTCATTGACCT	138
BruAb1_1305	GATGGCTAGCTGGCAATTCT	GTGGCAAAATCCGAATTGAT	105
BruAb1_2124	GAGCGAGAATATTGGCAAGG	ATGGCGATACGCTCAATTTC	105
BruAb2_0277	CTTTCGTCGGCTATCTCCTG	GCGGATCGAGATTGTAAAGC	138
BruAb2_0430	ATACTTGCCATCGCCTTGTC	AAAAGGTTGCAGGCAGAAGA	157
BruAb2_0692	GGTACTTTGCCGGTCGATTA	GCAGCGGTTACGATGAAAAT	185
BruAb2_1009	TGTGACGGCTGTAGGCTATG	CTGGGTCGGATCATGTTCTT	169
BruAb2_1128	CAAGAAACATTACGGCACCA	CACTCGTGAAGGTGGATGTG	146
BruAb1_0016	GACCTGCCAAAACCACTGAT	GGTAGCGGGTATCAGACCAA	147
BruAb1_0052	TCTCAGGCATGCAGAATCAG	CGGTATTGGCAGCAAAGTCT	149
BruAb1_0205	TCTTCAGCCAGCACTCTTCA	GTGGCATATTTCAGGCTCGT	180
BruAb1_0291	GGCCTTAATTCGCATGTCTC	CATGAACACCACTCCAGCAG	160
BruAb1_0427	TATCAAGTCCGGCAATGTCA	GAAGCGAAGGCGAGATTATG	174
BruAb1_0475	CAATCCTGATCGGCAATCTT	ATGGCAAAAACCAGAAGTGC	127
BruAb1_0505	AGTTTCCGTCCGCACAATAC	AGCTGCGAAGCGTAGAAGTC	152
BruAb1_0593	CGGCAGGAAGTCTCACGTAT	AACGAGGGCATCGAACTCTA	171
BruAb1_0603	GCTTGTCATGACGCTCTACG	TGATTTGCTATGCGAACGAG	90
BruAb1_1032	ATGTGCCGTCCTTTATCCAG	TCAAGATTTTCGGCCAATTC	149
BruAb1_1049	CGGAAACTGTTTTCCACCTC	TCGCCAATGTTTCCGTAGAT	97
BruAb1_1381	GCTTGCCGAAGTTTCTATCG	CCAGCCAAAGCTGTGATGTA	96

BruAb1_1442	GCAAACGCCCTGTCATCTAT	TCCGGTATATTGCCAGAAGG	152
BruAb1_1814	CGGCAGGAAGTCTCACGTAT	AACGAGGGCATCGAACTCTA	171
BruAb1_1926	CGGGGACTTCAGGAAAAACT	GGTTACGGATACGCAGCAAT	116
BruAb1_2148	AATTTTCCCATGGCGATGTA	TTCTTCGGATTTTCCGTGAC	197
BruAb2_0126	GCAAGGGCAGGTCTATTCAC	CCCCAACCTGTACACCATTC	182
BruAb2_0365	TCACATCCGGCATGATAGAA	GCCATTGGCATTGAAGAAAT	95
BruAb2_0572	GCGACAGAACAGACGATCAA	CTTCAAGGAAGCGATTTTCG	100
BruAb2_0753	CTCATCAGCCTTGCACAGAA	ATGAAATGGAACGACGAACC	185
BruAb2_0772	AAAGGATACGCCCTCCATCT	GCCGATGAAGCTGATAGACC	88
BruAb1_0099	CTTGTGGTGGAGGACGAAGT	GCGAGATTGACGTCCAGAAT	158
BruAb1_0993	ATTGTGGTGAGCTTGGGAAG	TTACCAGCAAAAGTGCGAAG	81
BruAb1_1330	GCGACAAGGAGAAGGTGAAG	TCGAAAGTCACGAGCACATC	130
BruAb1_1551	CTTATCCTCGGCACGACCTA	GCCGAAATTGGTGAAGTGAT	126
BruAb2_0061	CTGCTTGCGTGCATACTGAT	TAAATCCTGAGGCGACATCC	199
BruAb2_0699	TCTATCCGGCCTATGACCAG	GTTGACGGCATAACGGTCTT	190
BruAb2_0700	GATGTTGCGTGCCTATGATG	CATCGTGACGAGGGGATAGT	193
